# The Development, Application and Analysis of an Enhanced Recovery Programme for Major Oesophagogastric Resection

**DOI:** 10.1007/s11605-017-3363-8

**Published:** 2017-01-24

**Authors:** Timothy J. Underwood, F. Noble, N. Madhusudan, D. Sharland, R. Fraser, J. Owsley, M. Grant, J. J. Kelly, James P. Byrne, on behalf of the South Coast Cancer Collaboration-Oesophago-Gastric (SC -OG), A. Sansome, N. McGill, A. Cowan, C. Allan, D. Sparkes, L. Krivskiy, R. Chapman, J. Pointer, N. Perera, R. Devlin, S. Davies, R Simpson, M. Saunders, C. Sheikh

**Affiliations:** 1 Department of Surgery, University Hospital Southampton, Tremona Road, Southampton, Hampshire SO16 6YD UK; 2 Cancer Sciences Unit, Faculty of Medicine, University of Southampton, Somers Cancer Research Building, MP824, Southampton General Hospital, Southampton, Hampshire SO16 6YD UK

**Keywords:** Enhanced recovery, Oesophageal surgery, Surgery, Oesophagectomy, Gastrectomy

## Abstract

**Background:**

Enhanced recovery programmes improve outcomes in surgery, but their implementation after upper gastrointestinal resection has been limited. The aim of this study was to compare short-term outcomes for patients undergoing oesophagogastric surgery in an enhanced recovery programme (EROS).

**Methods:**

EROS was developed after a multidisciplinary meeting by multiple rounds of revision. EROS was applied to all patients undergoing major upper GI resection at a university teaching hospital in the UK from 20/9/13, with data reviewed at 18/09/15. EROS was assessed to identify predictors for compliance.

**Results:**

One hundred six patients underwent major upper GI resection including 81 oesophagectomies, 24 gastrectomies and 1 colonic interposition graft. Major complications (Clavien Dindo ≥3) occurred in 12 patients with 1 in-hospital death. Thirty-five patients (44%) were discharged on target day 8 of the EROS programme. Age and complications were independently associated with missing this discharge target.

**Conclusion:**

Enhanced recovery is feasible and safe after major upper gastrointestinal surgery.

**Electronic supplementary material:**

The online version of this article (doi:10.1007/s11605-017-3363-8) contains supplementary material, which is available to authorized users.

## Introduction

Enhanced recovery after surgery (ERAS) programmes have proven benefits in mortality and morbidity in a range of settings. ERAS programmes reduce patient length of stay (LOS) and reduce costs.^1^–^3^ The implementation of ERAS after upper gastrointestinal surgery has been limited by a number of factors. These include the historically high levels of mortality and morbidity after these types of operations and traditional surgical concerns regarding early feeding and anastomotic leak. The evidence for ERAS in oesophageal surgery is poor; in a recent systematic review, Findlay et al. identified only eight retrospective series with a total of 1127 patients. These were predominantly reports of single surgeon series and open resections.^4^ In some units, one surgeon but not others have embraced ERAS for oesophageal surgery^5^, and in other units, only the fittest patients have been included, making the overall benefits hard to establish.^6^

The use of minimally invasive oesophagectomy (MIO) is gaining momentum in the UK with 43.2% of resections being either totally minimally invasive or hybrid operations (laparoscopic abdomen and open chest) in the latest national audit.^7^ To date, very few reports have included patients treated with MIO within an enhanced recovery programme (ERP). The most recent systematic review and pooled analysis of enhanced recovery after oesophagectomy identified 27 cases of MIO in ERPs and 7 for MIO on conventional pathways.^8^

Therefore, the aims of this study were to develop, introduce and analyse an enhanced recovery pathway for all patients undergoing upper GI resection, including open, hybrid and totally minimally invasive oesophagectomy.

## Materials and Methods

### Enhanced Recovery After Oesophagogastric Surgery (EROS) Programme Development

The EROS programme was developed and implemented at University Hospital Southampton (UHS) NHS Foundation Trust, a single centre teaching hospital in the UK and a designated centre for oesophageal and gastric cancer surgery. A draft EROS programme was proposed by the surgical team and subjected to multidisciplinary professional review at a dedicated EROS development day (25/01/2012). This event was attended by Dr Donald Low (Virginia Mason Medical Centre, Seattle), who provided detail of the well-established programme at his centre, on which the UHS EROS programme was based.^9^ Multiple rounds of protocol revision were utilised to establish a programme that was considered as best practice and applicable to patients being treated in our centre by all key stakeholders (including surgeons, anaesthetists, specialist nurses, ward nurses, physiotherapists, occupational therapists, dieticians and patients (Supplemental document 1)). The fundamental components of the pathway involved pre, peri and post-operative elements including patient, carer and team expectation and education, preoperative carbohydrate loading, optimised anaesthesia, fluid and pain management, early and consistent mobilisation, timely removal of tubes and drains, early oral intake without the routine use of upper GI swallow studies and aggressive goal setting. Full details of the programme, including daily goals can be found in Supplemental document 2. The programme was initially designed for all patients who underwent a primary operation including oesophageal anastomosis (oesophagectomy or total gastrectomy), but in practice was used for all patients who underwent major surgery (including sub-total gastrectomy). The programme also included a previously published risk prediction tool for anastomotic leak and major complications after oesophageal surgery.^10^ The target time for discharge was set at post-operative day 8, with the goal of discharge to the patient’s home or usual place of residence without any increase in support. The EROS pathway was applied to all patients undergoing major oesophageal and gastric resections from 20/09/2013, with data presented up to 18/09/2015. There were no a priori exclusion criteria. Patients with carcinoma of the oesophagus or stomach were staged and considered for neoadjuvant therapy based on established protocols and underwent surgery as previously described.^11^

### Outcome Measures

Data recorded included demographics, tumour characteristics, type of resection, operative times (defined as knife-to-skin to wound closure), estimated blood loss (calculated from suction bottles and weighed swabs), critical care unit stay, overall hospital stay (defined as day of surgery until day of discharge), histopathologic analysis of the surgical specimen, morbidity and mortality. TNM-7 was used to report tumour stage. Pathologic tumour clearance (‘R’ status) was determined according the Royal College of Pathologists system (>1 mm clearance). Postoperative complications were graded according to the Clavien–Dindo classification^12^ and were recorded for the entire inpatient stay. Clavien–Dindo grades I and II represent minor complications, whereas grades III and IV represent major complications (grade III requires radiologic endoscopic or surgical intervention; grade IV indicates life-threatening complication requiring intensive care management). An anastomotic leak (AL) was confirmed by radiology (contrast-enhanced multi-detector CT scan or water-soluble contrast studies), endoscopy or during surgical exploration. Elements of EROS were recorded to provide a comprehensive assessment of compliance covering all aspects of the enhanced recovery programme.

### Statistics

Statistical analysis was performed with SPSS® version 21 (SPSS, Chicago, Illinois, USA). A *p* value <0.05 was considered statistically significant.

## Results

One hundred six consecutive patients underwent major upper GI surgery and a detailed analysis of this cohort is presented in Table 1, including oesophagectomies and gastrectomies shown separately.

**Table 1:** Patient, treatment and tumour demographics

Variable	Whole cohort	Oesophagectomy	Gastrectomy
Number of patients^b^	106	81	24
Median age (years)^a^	67 (26–87)	66 (40–82)	71 (26–81)
Sex ratio (M:F)	78:28	75:25	70:30
Pre-op median BMI^a^	27 (20–39)	27 (20–35)	26 (21–39)
ASA grade			
I	10 (9)	8 (10)	2 (8)
II	70 (66)	55 (68)	15 (63)
III	25 (24)	17 (21)	7 (29)
IV	1 (1)	1 (1)	
Neoadjuvant chemotherapy	49 (47)	35 (44)	14 (58)
Neoadjuvant chemoradiotherapy	25 (24)	25 (31)	
Surgery only	31 (30)	20 (25)	10 (42)
ypT or pT			
0	19 (18)	19 (24)	
IS/HGD	4 (4)	4 (5)	
1	12 (12)	9 (11)	3 (13)
2	9 (9)	8 (10)	1 (4)
3	47 (46)	38 (48)	9 (38)
4	12 (12)	2 (3)	10 (42)
ypN or pN			
0	58 (56)	53 (66)	5 (21)
1	14 (14)	11 (14)	3 (13)
2	19 (18)	11 (14)	8 (33)
3	12 (12)	5 (6)	7 (29)
ypM or pM			
0	96 (93)	76 (95)	20 (83)
1	7 (7)	4 (5)	3 (13)
Tumour type			
Adenocarcinoma	80 (76)	58 (72)	22 (92)
Squamous cell carcinoma	18 (17)	18 (22)	
Adenosquamous cell carcinoma	1 (1)	1 (1)	
Dysplasia	2 (2)	2 (2)	
Basaloid	1 (1)	1 (1)	
Leiomyoma/GIST	1 (1)		1 (4)
Neuroendocrine	1 (1)	1 (1)	
Benign ulcer	1 (1)		1 (4)

Values in parentheses are percentages unless indicated

^a^Values in parentheses are range

^b^Total cohort includes 1 colonic interposition

### Demographics

The median age was 67 years with a male predominance (74% male versus 26% female). Patients who underwent gastrectomy were older (median age 71 versus 66 years for oesophagectomy). The majority of patients had adenocarcinoma of the oesophagus or gastro-oesophageal junction and locally advanced disease (T3 in 56%) with lymph node metastasis (N1+ in 65%) on preoperative staging. Neoadjuvant therapy was used in 71% (chemotherapy in 49% and chemoradiotherapy in 24%).

### Operative Outcomes

Operative outcomes for the whole cohort and specifically for oesophagectomy and gastrectomy are presented in Table 2. There were 3 conversions to open procedures, all in the MIO group (*n* = 3/29 10%). The reasons for conversion were abdominal adhesions in 1 patient and in 2 patients the anaesthetist was unable to isolate the right lung at the beginning of the thoracic stage. Five operations required additional procedures, 2 lung resections (1 concurrent lung lesion of unknown aetiology and 1 concurrent primary lung cancer) and 3 splenectomy. Forty-eight patients (45%) had a feeding jejunostomy placed.

**Table 2:** Operative and surgical outcome data

Variable	Whole cohort	Oesophagectomy	Gastrectomy
Number of patients	106	81	24
Extra procedures	5 (5)	4 (6)	1 (4)
Feeding jejunostomy	48 (45)	46 (57)	1 (4)
Median duration of operation (min)^a^	329 (120–530)	340 (240–530)	210 (139–390)
Median blood loss (ml)^a^	250 (0–1892)	250 (0–1892)	150 (50–1400)
Median total length of stay (days)^a^	9 (4–90)	9 (6–90)	7 (4–31)
Median ITU stay^a^	0 (0–31)	0 (0–31)	0 (0–24)
Median HDU stay^a^	4 (0–13)	4 (0–13)	2 (0–4)
Conversions	3 (3)	3 (4)	0 (0)
Anastomotic leak	5 (5)	4 (5)	1 (4)
Chyle leak	8 (8)	7 (9)	1 (4)
Inpatient Clavien Dindo max grade			
0	42 (40)	27 (34)	15 (63)
1	7 (7)	5 (6)	2 (9)
2	45 (43)	39 (48)	5 (21)
3	3 (3)	3 (4)	0 (0)
4	8 (8)	6 (7)	2 (9)
5	1 (1)	1 (1)	0 (0)
Return to theatre	5 (5)	4 (5)	1 (4)
Cardiac complication	18 (17)	16 (20)	1 (4)
Respiratory complication	29 (27)	26 (32)	3 (13)
30 day mortality	0 (0)	0 (0)	0 (0)
90 day mortality	4 (4)	2 (3)	2 (9)
Readmission within 30 days of discharge	15 (14)	14 (17)	1 (4)
Median nodal yield^a^	24 (8–64)	26 (8–65)	30 (13–57)
Resection clearance			
R0	94 (89)	69 (85)	24 (100)
R1 (CRM)	12 (11)	12 (15)	0 (0)
R1 (Long)	0 (0)	0 (0)	0 (0)

Values in parentheses are percentages unless indicated

^a^Values in parentheses are range

A complete microscopic resection (R0) was achieved in 94 patients (89%), with a median lymph node yield of 24 (range 8–64).

Overall median length of stay was 9 days for all patients (range 4–90 days) and was 9 days for patients following oesophagectomy (range 6–90). All patients were discharged home (or to their usual place of residence) with no increase in the level of care provided except in 6 cases (5.6%) where increased support was required. Of these 6 patients, 4 experienced major complications and required readmission to intensive care (Clavien–Dindo grade 4). These patients had some of the longest lengths of stay. Of the other 2 patients, 1 had a pre-existing below-knee amputation and required a short-term increase in care and the other was an elderly man with no family support who suffered a post-operative chest infection. There was 1 in-hospital death (1%) at 59 days after a hybrid oesophagectomy. The patient died from multi-organ failure, after an anastomotic leak, having been treated preoperatively with high-dose steroids for concurrent lymphoma. There were three additional outpatient deaths: at 74 days after a hybrid oesophagectomy from rapidly progressive recurrent disease and at 88 and 89 days after palliative gastrectomy from disease progression; (90-day mortality: 4%).

Major complications (Clavien–Dindo; CD 3–4) occurred in 11 patients (11%). Anastomotic leak occurred in 5 patients (5%) and chyle leak in 8 patients (8%). Five patients (5%) required a return to theatre and 29 patients (27%) suffered respiratory complications, which were minor (CD <3) in the majority (20 patients). Fifteen patients (14%) were readmitted within 30 days of discharge.

A detailed analysis for oesophagectomies only is presented in Supplementary Tables 5 and 6.

### EROS Pathway Compliance

Table 3 shows all major elements of the Southampton EROS pathway assessed for compliance. The EROS pathway documentation was missing for 16 patients and 10 patients were taken off the pathway due to major complications. The target discharge date of 8 days after surgery was achieved in 44% of patients. In total 61% of patients achieved mobilisation of at least 25 m on post-operative day (POD) 1, including 30% of patients who achieved compliance with the POD 1 target mobilisation of 2 × 25 m walks. Similar compliance rates were observed for POD 2–5. Oral intake of fluids was established on POD 4 in 60% of patients and pureed diet was established by POD 6 in 59%. Nasogastric tubes were removed in the majority on POD 2 (54%) and in no patients was reinsertion required.

**Table 3:** Enhanced recovery after oesophagogastric surgery milestone compliance data

Variable	
Number of patients	106
Data analysed	80 (76)
Missing data	16 (15)
EROS discontinued	10 (9)
Patient and team expectations	
Preoperative patient information	68 (85)
Predicted discharge date recorded	3 (4)
Admission day of surgery	60 (75)
Post-operative destination Ward:HDU:ITU	1:70:9
Discharged home by POD 8	35 (44)
Analgesia	
PCA	79 (99)
Epidural	60 (75)
Mobilisation	
Hill-Rom bed utilised	53 (71)
POD 1	
2 walks	24 (30)
1 walk	25 (31)
4 h sat out	44 (56)
2 h sat out	14 (18)
POD 2	
3 walks	11 (14)
2 walks	29 (36)
1 walk	17 (21)
6 h sat out	49 (62)
3 h sat out	4 (5)
POD 3	
4 walks	13 (16)
3 walks	8 (10)
2 walks	29 (36)
1 walks	16 (20)
6 h sat out	49 (63)
3 h sat out	17 (22)
POD 4	
5 walks	5 (6)
4 walks	6 (8)
3 walks	11 (14)
2 walks	26 (32)
1 walk	19 (24)
6 h sat out	50 (64)
3 h sat out	15 (19)
POD 5	
6 walks	6 (8)
5 walks	1 (1)
4 walks	13 (16)
3 walks	12 (15)
2 walks	15 (19)
1 walk	8 (10)
6 h sat out	50 (68)
2 h sat out	8 (11)
Nutrition	
Preoperative carbohydrate loading	65 (81)
Day 1 step 1	80 (100)
Day 2 step 2	43 (54)
Day 4 step 3	48 (60)
Day 6 step 4	47 (59)
Tubes and drains	
TWOC day 4	53 (66)
NG out	
Day 2	43 (54)
Day 3	24 (30)
Day 4	10 (13)
Day 5+	3 (4)
Chest drains left removed by POD 4	54 (78)
Chest drains right removed by POD 4	39 (57)
Chest drains right removed by POD 5	45 (68)

Values in parentheses are percentages unless indicated

*POD* postoperative day

### Predictors of Discharge by Postoperative Day 8

A binary logistic regression analysis identified increasing age and post-operative complications as factors independently associated with missing the predefined target of discharge on POD8 (Table 4). Other factors including operative approach, perioperative outcomes, tumour stage, ASA grade and EROS targets were not associated with this discharge target.

**Table 4:** Binary logistic regression analyses of factors affecting discharge by POD 8

Variables		Univariate			Multivariate		
		HR	95% CI	*p* value	HR	95% CI	*p* value
Patient							
Age	Quartile 1 (25.5–59 years)	1	Ref		1	Ref	
	Quartile 2 (59–66.5 years)	1.589	0.475–5.310	0.452	1.648	0.428–6.354	0.468
	Quartile 3 (66.5–73 years)	1.901	0.564–6.410	0.300	2.007	0.507–7.942	0.321
	Quartile 4 (73–87 years)	3.870	1.085–13.812	*0.037*	7.190	1.616–31.995	0.010
Post-operative							
Maximum Clavien–Dindo inpatient complication	No (0)	1	Ref				
	Minor (1–2)	4.050	1.708–9.602	*0.001*	5.257	1.774–15.575	0.003
	Major (3–5)	19.800	2.324–168.661	*0.006*	28.381	2.946–273.370	0.004

### Pre-EROS Experience

In the calendar year preceding the start of EROS, 72 consecutive patients underwent major upper GI resections (51 oesophagectomies). The overall median length of stay was 11 days (range 4 to 55 days) and 10 days for oesophagectomies (range 6 to 43 days). Major complications occurred in 18% and there were 3 deaths.

## Discussion

This study demonstrated that EROS could be applied to all patients who underwent major upper GI surgery with good outcomes. Enhanced recovery was safe and effective after oesophageal surgery and delivered low levels of morbidity and short lengths of stay.

These findings are in line with results from other surgical disciplines. For instance, in colorectal surgery ERAS halves morbidity and significantly reduces length of stay.^13^ Published results for enhanced recovery after oesophageal surgery have shown more modest improvements, possibly due to reluctance by the clinical teams to progress patients quickly for fear of significant, life-threatening complications. The findings of this study support the application of enhanced recovery for all upper GI patients undergoing major resection, with relatively aggressive targets. No major differences in outcome between different surgical approaches were observed, but the study was not designed to explicitly address this question.

The development of the EROS programme took into account the infrastructure and resources of the institute where it was introduced, meaning that it may not be applicable to different hospitals and healthcare systems.^14^ This could also be considered as a benefit of the programme, as EROS contains the fundamental elements of enhanced recovery tailored to the local environment. Other centres wishing to introduce enhanced recovery can be reassured that adapting existing programmes for use in their own hospitals is possible and leads to good outcomes. Our experience with EROS suggests that the multidisciplinary team begin to see the programme as the default pathway for all patients undergoing major surgery. Anecdotally, mobilisation of all patients has improved and the “STEP” system (supplementary document 1) introduced for oral intake is now applied to all upper GI patients. This has the advantage of removing variation for the junior medical and nursing staff whose shift patterns change regularly.

The study had a number of strengths. The cohort was consistent with contemporary clinical practice; the majority of patients had locally invasive, node positive oesophageal adenocarcinoma treated with neoadjuvant therapy. The surgeons involved had significant experience with minimally invasive oesophagectomy^11^ meaning that the applicability of the results for MIO have not been biased by a “learning-curve” effect. Morbidity data was comprehensively collected by a dedicated data manager and is reported using a validated system.

Overall outcomes are satisfactory and comparable with data from the UK National Oesophagogastric Cancer Audit (NOGCA)^7^ and previously published series.^9^ An improvement in overall length of stay was observed when compared to the year preceding the introduction of the programme (9 versus 11 days) and our previously published comparison of open and minimally invasive oesophagectomy (9 versus 12 days).^11^ However, it is important to note that the benefit for oesophagectomies alone when comparing the EROS time period with the preceding year was only 1 day (9 versus 10 days). A potentially more clinically relevant improvement was observed for gastrectomies (7 versus 11 days), but the number of gastrectomies in the EROS cohort was modest (*n* = 24).

Whilst in this study, we have made no attempt to provide a detailed “before and after” analysis of EROS, major morbidity of 10% compares favourably with our experience (18%) in the year preceding the introduction of the programme and with other published series of oesophagectomies performed both on conventional care pathways and within ERPs.^5^, ^9^, ^11^ Reduced morbidity for patients in EROS confirms previous reports from all branches of surgery and may represent the major benefit of enhanced recovery. This is particularly important for oesophageal surgery that has witnessed a dramatic reduction in mortality in recent years, but has struggled to make in-roads into relatively high complications rates.^7^

A reduction in pulmonary complications has been reported as a significant benefit of both enhanced recovery and MIO.^15^, ^16^ In this series, respiratory complications were observed in 27% of patients. This apparently high level of respiratory complications is in keeping with patients treated on a conventional pathway (29.1%)^8^ but includes patients with relatively minor deviations to the clinical course (Clavien–Dindo 1 and 2) in the vast majority. When only major respiratory complications are considered, the incidence falls to 6.6%.

Considerable concern exists within the surgical community regarding gastric conduit decompression and the relevance of drainage procedures. No pyloroplasties or other drainage procedures were performed in this series and there were no instances of acute conduit distension. It appears feasible and safe to remove nasogastric tubes and recommence oral intake early in the post-operative course (POD 2) without the routine use of upper GI swallow studies.

We observed a significant number of readmissions within 30 days of discharge (14%). This may reflect an overly ambitious policy of discharge, but is also related to an “open-door” attitude towards discharged patients and the local geography (the catchment area includes the Isle of Wight and the Channel Islands). Our experience suggests that rather than being viewed as a negative outcome, readmission should be expected in a relatively small proportion of patients as part of the enhanced recovery programme. Further community support for patients and their carers will be required to reduce readmissions and will be a focus for pathway development.

Reporting of compliance data in ERPs is poor^17^ and patients with complications are often removed from ERPs. This makes it impossible to determine the efficacy of different aspects of the ERP and their relative effects on outcome.^14^ In an attempt to address this, compliance with the major agreed components of EROS was documented and analysed. Age and complications defined by the Clavien–Dindo classification were independently associated with missing the predefined discharge target of 8 days. These factors may not be surprising but offer potential ways to improve the pathway. For example, patients of advanced age should be highlighted in the neoadjuvant or preoperative setting as potentially requiring prehabilitation interventions or increased packages of care to enable them to continue their rehabilitation, in the community. In addition, the focus should shift to preventing minor as well as major complications and this will require a whole multidisciplinary team approach.

In this study, compliance with target mobilisation was poor. However, this probably reflects overambitious target setting. The advantage of such target setting is that >60% of patients walked >25 m on POD 1 and an improvement in mobilisation quantity and distance was observed for each post-operative day. Consistent mobilisations early in the post-operative course proved labour intensive and difficult to achieve and we may have overestimated the availability of local resources to deliver this. Future efforts will be focussed on improving compliance in this area and will depend upon the continued development of the multidisciplinary team.

## Conclusion

The experience described in this study with the introduction of EROS demonstrates that enhanced recovery is feasible and safe after major upper gastrointestinal surgery.

## Electronic Supplementary Material

Below is the link to the electronic supplementary material.

ESM 1 (DOC 95 kb)

ESM 2 (DOC 70 kb)

ESM 3 (DOC 722 kb)

ESM 4 (DOC 174 kb)
