# Supplementary material for: The Development, Application and Analysis of an Enhanced Recovery Programme for Major Oesophagogastric Resection
Source: J Gastrointest Surg. 2017 Jan 24;21(4):614–21. doi: 10.1007/s11605-017-3363-8 (PMC5359364; doi:10.1007/s11605-017-3363-8)
Supplement: Supplementary file 1 — (DOC 95 kb) [file 11605_2017_3363_MOESM1_ESM.doc]

| **Table 5 Patient, treatment and tumour demographics for oesophagectomies.** | | | | | |
| --- | --- | --- | --- | --- | --- |
| **Variable** |  | **IVL** | **MIO-2** | **Hybrid** | ***p-value*** |
| **Number of patients** |  | 33 | 29 | 18 |  |
| **Median Age (years) *** |  | 66 (49-82) | 66 (41-80) | 69 (46-81) | 0.384† |
| **Sex Ratio (M:F)** |  | 26:7 | 22:7 | 13:5 | 0.870† |
| **Pre-op median BMI *** |  | 27 (21-34) | 26 (20-34) | 27 (20-35) | 0.589† |
| **ASA Grade** |  |  |  |  | 0.127† |
|  | **I** | 5 (15) | 3 (10) | 0 (0) |  |
|  | **II** | 24 (73) | 17 (59) | 13 (72) |  |
|  | **III** | 3 (9) | 9 (31) | 5 (28) |  |
|  | **IV** | 1 (3) | 0 (0) | 0 (0) |  |
| **Pre-treatment Stage T** |  |  |  |  | 0.349† |
|  | **0** | 0 (0) | 2 (7) | 2 (11) |  |
|  | **1** | 0 (0) | 1 (3) | 0 (0) |  |
|  | **2** | 7 (21) | 7 (24) | 5 (28) |  |
|  | **3** | 25 (76) | 15 (52) | 11 (61) |  |
|  | **4** | 1 (3) | 4 (14) | 0 (0) |  |
| **Pre-treatment Stage N** |  |  |  |  | 0.198† |
|  | **0** | 8 (24) | 11 (38) | 5 (28) |  |
|  | **1** | 21 (64) | 18 (62) | 10 (56) |  |
|  | **2** | 4 (12) | 0 (0) | 3 (17) |  |
| **Pre-treatment Stage M** |  |  |  |  | 0.491† |
|  | **M0** | 32 (97) | 29 (100) | 18 (100) |  |
|  | **M1** | 1 (3) | 0 (0) | 0 (0) |  |
| **Neoadjuvant Chemotherapy** | | 19 (58) | 13 (46) | 3 (17) | 0.266† |
| **Neoadjuvant Chemoradiotherapy** | | 8 (24) | 8 (29) | 8 (44) |
| **Surgery only** | | 6 (18) | 7 (25) | 7 (39) |
|  |  |  |  |  |  |
| **ypT or pT** |  |  |  |  | 0.784 † |
|  | **0** | 8 (24) | 6 (21) | 5 (29) |  |
|  | **IS/HGD** | 0 (0) | 3 (10) | 1 (5.9) |  |
|  | **1** | 3 (9) | 4 (14) | 2 (12) |  |
|  | **2** | 2 (6) | 4 (14) | 2 (12) |  |
|  | **3** | 18 (55) | 12 (41) | 7 (41) |  |
|  | **4** | 2 (6) | 0 (0) | 0 (0) |  |
| **ypN or pN** |  |  |  |  | 0.846 † |
|  | **0** | 20 (61) | 20 (69) | 12 (71) |  |
|  | **1** | 6 (18) | 4 (14) | 1 (6) |  |
|  | **2** | 6 (18) | 2 (7) | 3 (18) |  |
|  | **3** | 1 (3) | 3 (10) | 1 (6) |  |
| **ypM or pM** |  |  |  |  | **0.028** † |
|  | **0** | 33 (100) | 26 (86) | 17 (100) |  |
|  | **1** | 0 (0) | 4 (14) | 0 (0) |  |
| **Tumour Type** |  |  |  |  | 0.087 † |
|  | **AC** | 25 (76) | 17 (59) | 15 (83) |  |
|  | **SCC** | 7 (21) | 9 (31) | 1 (6) |  |
|  | **AS** | 0 (0) | 1 (3) | 0 (0) |  |
|  | **Dysplasia** | 0 (0) | 1 (3) | 1 (6) |  |
|  | **Basaloid** | 0 (0) | 1 (3) | 0 (0) |  |
|  | **Leiomyoma** | 0 (0) | 0 (0) | 1 (6) |  |
|  | **Neuroendocrine** | 1 (3) | 0 (0) | 0 (0) |  |

Kruskal Wallis Test † Values in parentheses are percentages unless indicated. *Values in parentheses are range
