# Supplementary material for: The Development, Application and Analysis of an Enhanced Recovery Programme for Major Oesophagogastric Resection
Source: J Gastrointest Surg. 2017 Jan 24;21(4):614–21. doi: 10.1007/s11605-017-3363-8 (PMC5359364; doi:10.1007/s11605-017-3363-8)
Supplement: Supplementary file 2 — (DOC 70 kb) [file 11605_2017_3363_MOESM2_ESM.doc]

| **Table 6 Operative and surgical outcome data for oesophagectomies** | | | | | |
| --- | --- | --- | --- | --- | --- |
| **Variable** |  | **IVL** | **MIO-2** | **Hybrid** | ***p-value*** |
| **Number of patients** |  | 33 | 29 | 18 |  |
| **Extra Procedures** |  | 1 (3) | 1 (3) | 1 (6) | 0.852 † |
| **Feeding jejunostomy** |  | 27 (82) | 7 (24) | 11 (61) | **<0.0001 †** |
| **Median duration of operation (min) *** | | 331 (240-513) | 346 (254-530) | 340 (270-390) | 0.745 † |
| **Median blood loss (ml) *** |  | 350 (100-1892) | 175 (60-900) | 226 (0-800) | **<0.0001 †** |
| **Median total length of stay (days) *** | | 9 (6-90) | 8 (6-52) | 10 (7-59) | 0.474 † |
| **Median ITU stay *** |  | 0 (0-17) | 0 (0-25) | 0 (0-28) | 0.783 † |
| **Median HDU stay *** |  | 4 (1-11) | 4 (0-13) | 4 (0-6) | 0.378 † |
| **Conversions** |  | n/a | 3 (10) | 0 (0) | n/a |
| **Anastomotic Leak** |  | 0 (0) | 2 (7) | 1 (6) | 0.330 † |
| **Chyle Leak** |  | 4 (12) | 1 (3) | 2 (11) | 0.450 † |
| **Inpatient Clavien Dindo Max grade** | |  |  |  | 0.596 † |
|  | **0** | 12 (36) | 9 (31) | 6 (33) |  |
|  | **1** | 3 (9) | 2 (7) | 0 (0) |  |
|  | **2** | 16 (49) | 14 (48) | 9 (50) |  |
|  | **3** | 1 (3) | 2 (7) | 0 (0) |  |
|  | **4** | 1 (3) | 2 (7) | 2 (11) |  |
|  | **5** | 0 (0) | 0 (0) | 1 (6) |  |
| **Return to Theatre** |  | 0 (0) | 2 (7) | 1 (6) | 0.330 † |
| **Cardiac complication** |  | 6 (18) | 5 (17) | 5 (28) | 0.645 † |
| **Respiratory complication** |  | 10 (30) | 10 (35) | 5 (28) | 0.881 † |
| **30 day mortality** |  | 0 (0) | 0 (0) | 0 (0) | 1.000 † |
| **90 day mortality** |  | 0 (0) | 0 (0) | 2 (11) | **0.031 †** |
| **Readmission** |  | 8 (24) | 4 (14) | 2 (11) | 0.406 † |
|  |  |  |  |  |  |
| **Median Nodal Yield *** |  | 24 (8-64) | 28 (12-62) | 27 (10-65) | 0.269 † |
| **Resection Clearance** |  |  |  |  | 0.601 † |
|  | **R0** | 30 (91) | 24 (83) | 15 (83) |  |
|  | **R1 (CRM)** | 3 (9) | 5 (17) | 3 (17) |  |
|  | **R1 (Long)** | 0 (0) | 0(0) | 0 (0) |  |

Kruskal Wallis test † Values in parentheses are percentages unless indicated. *Values in parentheses are range
