# Supplementary material for: The Development, Application and Analysis of an Enhanced Recovery Programme for Major Oesophagogastric Resection
Source: J Gastrointest Surg. 2017 Jan 24;21(4):614–21. doi: 10.1007/s11605-017-3363-8 (PMC5359364; doi:10.1007/s11605-017-3363-8)
Supplement: Supplementary file 3 — (DOC 722 kb) [file 11605_2017_3363_MOESM3_ESM.doc]

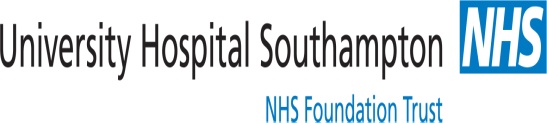


**Enhanced Recovery Following Oesophago-gastric Surgery (EROS) Care Pathway**

| **Version:** | **4** |
| --- | --- |
| **Approval Committee:** | **Surgical Governance Group** |
| **Date of Approval:** | **Nov 2014** |
| **Ratification Committee (Level 1 documents):** | **N/A** |
| **Date of Ratification (Level 1 documents):** | **N/A** |
| **Signature of ratifying Committee Group/Chair (Level 1 documents):** | **N/A** |
| **Lead Job Title of originator/author:** | **James Byrne, Consultant Surgeon** |
| **Name of responsible committee/individual:** | **Surgical Governance Group** |
| **Date issued:** | **06 Nov 2014** |
| **Review date:** | **06 Nov 2015** |
| **Target audience:** | Multidisciplinary team looking after surgical upper GI patients. |
| **Key words:** | **EROS, Enhanced Recovery** |
| **Main areas affected:** | **Surgical HDU, GICU, E5, Day Surgery Unit, Recovery. Preassessment Clinic** |
| **Summary of most recent changes:** | **Complete review and rewrite.** |
| **Consultation:** | **All members of the multidisciplinary team** |
| **Equality Impact Assessments completed and policy promotes equity** | **Yes** |
| **Number of pages:** | **33** |
| **Type of document:** | **Level 2** |
| **Is this document to be published in any other format?** | **Available through staffnet only** |

The Trust strives to ensure equality of opportunity for all, both as a major employer and as a provider of health care. This (insert document name) has therefore been equality impact assessed to ensure fairness and consistency for all those covered by it, regardless of their individual differences, and the results are available on request.


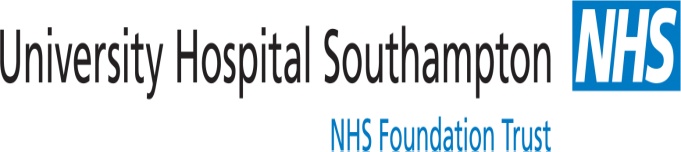


OESOPHAGO-GASTRIC RESECTION SURGERY

ENHANCED RECOVERY AFTER OESOPHAGO-GASTRIC SURGERY

(EROS)

CARE PATHWAY

| Patient Label |
| --- |

**Consultant:**

**Date of Admission:**

| All staff signing for care within this pathway please record overleaf your full name and a sample of your initial signature **(this is a legal requirement).**  Record any variances from management goals.  Upper GI HO: bleep 1551  Upper GI SHO: bleep 2150  Upper GI SpR: bleep 1117  Upper GI Nurse Specialist: 07768 447611 |
| --- |

| EROS Care Pathway | (Patient Label) | |
| --- | --- | --- |
| **HOW TO USE THIS CARE PATHWAY**  Care Pathways (CPs) are being used to make sure that the care we offer is: | | |
| - Of the highest quality. - The best we can deliver | | - Evidence-based - Efficient |
| The EROS Focus Group has discussed and agreed the care that is best for a typical patient that meets the criteria for this CP. The CP is therefore a guideline of the best multidisciplinary care for the EROS patient. Remember, however, that every patient is an individual and that this CP is NOT a substitute for your clinical judgement and expertise. You should therefore use it as follows: | | |
| 1. Complete the sample initial box below.  2. Look at what is planned for the patient today as set out in the CP and decide if this is appropriate to your patient. If so, deliver the care and initial in the box next to the activity.  *(NB Some sections require the actual times to be recorded when*  *something happens to the patient so be sure to note these times.)*  3. If the planned care is not appropriate for your patient, then you need to record this as a “variance” (V) in the initial box. The reason for the variance should then be documented on the additional notes section on each day, and discussed with the EROS Co-ordinator or Medical team.  4. This document must be completed in conjunction with the Daily assessment care plan. | | |

RECORD OF SIGNATURES

Please enter details if you have initialled in any part of this document.

| NAME (Print) | | **SIGNATURE** | | **INITIALS** | **JOB TITLE** |  |
| --- | --- | --- | --- | --- | --- | --- |
|  | |  | |  |  |  |
|  | |  | |  |  |  |
|  | |  | |  |  |  |
|  | |  | |  |  |  |
|  | |  | |  |  |  |
|  | |  | |  |  |  |
|  | |  | |  |  |  |
|  | |  | |  |  |  |
|  | |  | |  |  |  |
|  | |  | |  |  |  |
|  | |  | |  |  |  |
|  | |  | |  |  |  |
|  | |  | |  |  |  |
|  | |  | |  |  |  |
|  | |  | |  |  |  |
|  | |  | |  |  |  |
|  | |  | |  |  |  |
|  | |  | |  |  |  |
|  | ABBREVIATIONS | |  | | | |
|  | APTR | | Activated Partial Thromboplastin Ratio | | | |
|  | AXR | | Abdominal X-ray | | | |
|  | BM | | Blood Monitoring (glucose) | | | |
|  | BMI | | Body Mass Index | | | |
|  | B.P | | Blood Pressure | | | |
|  | CXR | | Chest X-ray | | | |
|  | DVT | | Deep Vein Thrombosis | | | |
|  | ECG | | Electrocardiogram | | | |
|  | ECHO | | Echo cardiogram | | | |
|  | EROS | | Enhanced Recovery After Oesophageal Surgery | | | |
|  | FBC | | Full Blood Count | | | |
|  | G&S | | Group and Save | | | |
|  | HMR | | Home Medicines Record | | | |
|  | IPC | | Intermittent Pneumatic Compression boot (Flowtron) | | | |
|  | INR | | International Normalised Ratio | | | |
|  | IV | | Intravenous | | | |
|  | IVI | | Intravenous Infusion | | | |
|  | K+ | | Potassium | | | |
|  | LFT’s | | Liver Function Tests | | | |
|  | LMP | | Last Menstrual Period | | | |
|  | Jej | | Jejunostomy | | | |
|  | MEWS | | Modified Early Warning System | | | |
|  | MDT | | Multidisciplinary Team | | | |
|  | MIO | | Minimally Invasive Oesophagectomy | | | |
|  | MRSA | | Methicillin Resistant Staphylococcus Aureus | | | |
|  | MSU | | Mid Stream Urine. | | | |
|  | MUST | | Malnutrition Universal Screening Tool | | | |
|  | N | | No | | | |
|  | N&V | | Nausea and vomiting | | | |
|  | Na | | Sodium | | | |
|  | N/A | | Not applicable | | | |
|  | NAD | | Nothing Abnormal Detected | | | |
|  | NBM | | Nil By Mouth | | | |
|  | Nocte | | Night | | | |
|  | NG | | Nasogastric Tube | | | |
|  | NJ | | Nasojejunal Tube | | | |
|  | 02 | | Oxygen | | | |
|  | OT | | Occupational Therapist | | | |
|  | OPA | | Out Patient Appointment | | | |
|  | PCA | | Patient Controlled Analgesia | | | |
|  | PGD | | Patient Group Direction | | | |
|  | POD's | | Patients Own Drugs | | | |
|  | PR | | Per Rectum | | | |
|  | PRN | | As Required | | | |
|  | QDS | | Four times a day | | | |
|  | RESP’s | | Respirations | | | |
|  | ROS | | Removal of Sutures | | | |
|  | SAT’s | | Saturations | | | |
|  | TDS | | Three times a day | | | |
|  | TTO’s | | Tablets to take out. | | | |
|  | TWOC | | Trial Without Catheter | | | |
|  | U’s & E’s | | Urea and Electrolytes | | | |
|  | V | | Variance | | | |
|  | VIP | | Visual Infusion Phlebitis Score | | | |
|  | Y | | Yes | | | |

| EROS Care Pathway Pre-assessment  Medical Checklist | (Patient Label) |
| --- | --- |
| DATE: |
| **PLEASE NOTE:** *All Trust Pre-assessment documents should be used in conjunction with this checklist*. | |

| **PLANNED SURGERY:** | | | | |
| --- | --- | --- | --- | --- |
| **PLANNED DATE OF ADMISSION:** | | | **CONSULTANT:** | |
| **INVESTIGATIONS:** (Tick when requested)  *(Essential) (if required)* | | | | |
| - **FBC** |  | - **Chest X-ray** | |  |
| - **U+E’s** |  | - **Echo** | |  |
| - **MRSA Screen** |  | - **Pulmonary Function Tests** | |  |
| *** Clotting** |  | - **Blood Gasses** | |  |
| - **G&S** |  | *** Other (please specify)** | |  |
| - **ECG** |  |  | |  |
|  | | | | |
| **COMPLETE VENOTHROMBOEMBOLISM RISK ASSESSMENT** | | | |  |
| **COMMENCE DRUG CHART** | | | |  |
| **Provide via PDG 2 x 200ML CARTON “PreOp”, CARB LOADING DRINK.**  **05:30 ON DAY OF SURGERY. (Patient to take home). - NOT for Type 1 DM, give to type 2 DM with caution** | | | |  |

| **ANAESTHETIC REVIEW** | | |
| --- | --- | --- |
| **CONSULTANT ANAESTHETIST NAME** |  | |
| **INVESTIGATIONS REQUESTED** |  | |
| **OUTCOME OF PREASSESSMENT** | **FIT FOR SURGERY**  **Y / N** | **NOT FIT FOR SURGERY**  **Y / N** |
|  |  | **REASON FOR DECISION;** |

**Completed By: Time:**

| **EROS Care Pathway**  Pre-assessment  Nursing Checklist | | (Patient Label) | | | | |
| --- | --- | --- | --- | --- | --- | --- |
| DATE: | |
| PATIENT EDUCATION. | | | Relative/ Carer present: YES / NO | | | |
| Introduce EROS program.Patient information booklet given. | | | | | |  |
| Ward routines explained, including ward visiting times. | | | | | |  |
| Postoperative pain control explained. | | | | | |  |
| Patient mobility targets discussed. Mobility targets defined with patient depending on exercise tolerance.   | **POOR** | **MODERATE** | **UNLIMITED** | | --- | --- | --- | | 10m | 50m | 100m | | | | | | | |
| 2 x“PreOp”(200ml) drinks given to patient to drink at 05.30 hrs on morning of surgery. | | | | | |  |
| Patient advice given regarding immediate postoperative diet and nutrition. Refer to patient information booklet. | | | | | |  |
| **Planned thromboprophylaxis explained.** | | | | | |  |
| **REFERRALS (If required)** | | | | | |  |
| **Dietician (If MUST score greater than or equal to 2, or if patient has complex nutritional concerns.)** (Via Equest) | | | | | | **YES / N/A** |
| **Nutrition Score – Need for pre operative jej feeding – discussed with patient.** | | | | | | **YES** |
| **Is patient likely to need health and social care support on discharge?**  If yes please document anticipated needs and complete Section 2 form  **Section 2 completed** | | | | | | YES / NOYES / N/A |
| **Contact Denise Whittaker trials coordinator for consideration of inclusion into portfolio studies (OG trials). Ext. 3027 Mob. 07584206918** | | | | | | YES / NO |
| Predicted Discharge Date: | | | | | | |
| **Type of accommodation** | **Does Patient Live alone?** | | | **Current Care Package** | | |
|  |  | | |  | | |
| **Discharge arrangements discussed and agreed with patient/ relative.** | | | | | YES/NO | |
| **Patient asked to make Pre 11 am transport arrangements for day of discharge.** | | | | | YES/NO | |

**Nurse completing assessment:**

**Signature: Time:**

| **PRE-ASSESSMENT**  **Clerking** | PATIENT STICKER |
| --- | --- |

Date

TCI date (if known)

Age

Operation

Surgeon

Past surgical / anaesthetic history

Past medical history (Other than below)

MI or IHD

HT

CVA or TIA

Diabetes

DVT or PE (patient or family)

Peripheral vascular disease

Asthma or COPD

GI ulcer or bleed

Epilepsy

| **PRE-ASSESSMENT**  **Clerking** | PATIENT STICKER |
| --- | --- |

DRUG HISTORY SHEET

| **Drug History from** | Patient’s own drugs | Repeat Rx list | Patients’s list |
| --- | --- | --- | --- |
| Contact GP | Relative/carer | Dosette/nomad | other |

Allergies

| **Drug/Allergen** | **Reaction** | **When/comments** |
| --- | --- | --- |
|  |  |  |

Current medication

| **Drug name** | **Dose** | **Frequency (and time)** | **Pre-op plan**  **c = continue s = stop** |
| --- | --- | --- | --- |
|  |  |  |  |

*****Any herbal remedies, inhalers, over-the-counter medicines**

Steroids

| **Preparation** | **Dose/frequency** | **Indication** | **Course** | **Date last taken** |
| --- | --- | --- | --- | --- |
|  |  |  | Short/ on-going |  |

Warfarin

| **Indication** | **Usual dose** | **Target INR** | **Last INR** | **Pre-op plan** |
| --- | --- | --- | --- | --- |
|  |  |  |  |  |

If DVT or PE please obtain details of history: when, how many events, ppt factors

Diabetes: Yes / No Peri-operative plan: Sliding scale

Omit medication and monitor BM

Continue medication

Advised to bring drugs on admission 

| **PRE-ASSESSMENT**  **Clerking** | PATIENT STICKER |
| --- | --- |

Smoking: /day Alcohol: /week

Occupation:

Social history: Lives alone Lives with spouse/family warden controlled flat

Rest home nursing home

Lives in: house with stairs bungalow other:

**Functional Enquiry**

Exercise tolerance: Limited by:

| CVS: chest pain  Orthopnoea or PND pillows used?  Palpitations, dizzy spells, collapse  Claudication distance?  Resp: cough or wheeze?  Regular infections needing antibiotics how often?  Haemoptysis | | |
| --- | --- | --- |
| Abdo: wt loss  Acid indigestion / reflux  Urol: regular UTI or haematuria  Neuro: numbness or tingling  Regular headaches  Blackouts or epileptic fit  Neck or back pain or injury  Other: thyroid problems | | | **Bleeding history**  Easy bruising   Bleed after surgery?   Bleed after dental work?   Bleed after childbirth?   Epistaxis   Menorrhagia   Family hx bleeding problems?  | | --- | |
| **PRE-ASSESSMENT**  **Clerking** | PATIENT STICKER | |

|  | **EXAMINATION**  **Hands:** clubbing pale cool warm tremor  **nails:** normal Nicotine stains arthritis capillary refill <2 sec: Yes/No  **Excess bruising:** Yes/No  **Radial pulse:** rhythm: regular irregular  **Neck:** scars:  JVP: not seen seen: normal raised cm  **Eyes** pallor sclera white pupils equal  **Mouth**  dentition good reasonable poor  **Airway examination**  Neck extension good reasonable poor  Mouth opening 2 finger breadths less than 2 finger breadths  Upper teeth to lower teeth: normal overhanging upper teeth  Mallampati 1 = see who uvula 3 = uvula not seen, but can see soft palate  2 = see part of uvula 4 = only roof of mouth visible  Inspect short of breath in clinic? Not slightly markedly  Breathing pattern normal abnormal:  Shape of chest normal hyperexpanded P excavates  Expansion: symmetrical asymmetrical:  Palpation expansion: symmetrical asymmetrical:  Apex beat 5^th^ ISC MCL displaced not felt  Percussion equal and resonant other:  Auscultation wheezes xx crackles: fine.. coarse…  Heart sounds normal other  Ankle swelling: none pitting oedema to:  **Investigations**  FBC INR Echo ECG findings:  U&E TFT PFT  HbA1C Coag G&S If AF: CHADS_2_ score =  **Summary:** No issues  Notes review  Anaes review  **Signature:** |  |
| --- | --- | --- |
| Weight  BMI  P  BP  Sp0_2_ |  |  |
|  |  | BMI = possible difficult airway?  **Yes**  **No** |
|  |  |  |

| **PRE-ASSESSMENT**  **Outcome Sheet** | PATIENT STICKER |
| --- | --- |

**Pre Assessment Date: Admission Date:**

**Pre Assessment Nurse: Consultant:**

**OUTCOME: FIT / UNFIT / DNA**

**Operation:**

| Issues arising from history taking and physical examination.  **Has the patient been examined? Yes / No**  **Has the patient been seen by the Anaesthetist Yes / No (see anaesthetic sheet)** | | | |
| --- | --- | --- | --- |
|  |  | | |
| To be done on admission: | | | |
|  |  | | |
| Admit DOS? Yes/No Admit day before Op? Yes/No  Other…………………………………………………………………………………………………………………………………………………………… | | | |
|  | |  | |
| Was the patient chaperoned? Yes / No / Declined  Smoking cessation advice Yes / No / NA  VTE Risk assessment Yes / No  Bariatric form Yes / No / NA  CJD form Yes / No  Hibiscrub and info Yes / No  Social services Yes / No / NA  Patient info leaflet Yes / No  Medication instructions Yes / No / NA | | | Pregnancy advice given Yes / No  Anaesthetic booklet Yes / No  VTE patient information Yes / No  Rivoroxaban leaflet Yes / No  Clexane pack given Yes / No  PROMs form given Yes / No  Surgical wound leaflet Yes / No |

**Blood results seen and accepted Yes / No…………………………………………………………….. (Signature)**

**Antibody screen confirmed as negative Yes / No / N/A………………………………………………….. (Signature)**

**MSU sent Yes / No / N/A MRSA +ve Yes / No**

**Result of MSU…………………………………**

| **ER PATHWAY**  **INPATIENT STAY DOCUMENTATION** | PATIENT STICKER |
| --- | --- |

**Please use the following pages to document all aspects of the patients care.**

**This is a multidisciplinary document; no additional medical or nursing notes are required.**

| EROS Care Pathway  On Admission | (Patient Label) | |
| --- | --- | --- |
| **DATE:** |
| PLANNED SURGERY: | | **Cons:** |
| **PLEASE NOTE:** Nursing Risk Assessment documents including SIRFIT, Braden and MUST scores should be completed in conjunction with this document. | | |

| **ADMISSION CHECKLIST** | ***(Initial)*** |
| --- | --- |
| Patient reviewed by surgical team. |  |
| Investigation results available – Clinic and MDT letters printed and in notes. |  |
| G&S sample taken within last week. |  |
| Blood results checked and repeated if necessary. |  |
| Drug chart written. |  |
| Theatre Consent Signed. |  |
| Trial Consent Signed. |  |
| Anaesthetic assessment. |  |

| **NURSING CHECKLIST** | | |
| --- | --- | --- |
| ***Admission Data*** | | |
| Patient orientated to ward.  *(Include access to nutritional drinks and washing facilities)* |  | |
| VALUABLES  | Glasses | Hearing Aid | Contact Lenses | Dentures | | --- | --- | --- | --- | |  |  |  |  |   *N.B. Ensure adherence to Trust Valuables Policy.* | | |
| Name bands and allergy band applied to patient |  | |
| *Nutrition* | | |
| - Normal Diet until 22.00 day before surgery. - Clear water until 2 hrs before surgery. - 2 x “PreOp” Carb loading drinks given to all EROS patients at: 05.30 - NBM from 06.00 |  | |
| **Thromboprophylaxis** | | |
| Thromboprophylaxis prescribed as per protocol. (Circle) | **YES** | **NO** |
| Antiembolic stockings fitted. | **YES** | **NO** |
| **Patient Education** | | |
| Patient given copy of “Patient Targets” booklet. Reiteration of patient’s role in recovery process including deep breathing, mobilisation and nutrition and hydration. |  | |

Completed by: Time:

| EROS Care Pathway  Day of Surgery  07.00am- 06.59am | (Patient Label) |
| --- | --- |
| DATE: |
| **PLEASE NOTE:** Trust pre-operative checklist should be used in conjunction with this document. | |

| **Urine Output Target Weight (Kg) =**  **0.33 ml/kg/hr =**  **Over 4 hrs =**  **Document above on Fluid Balance chart.** |
| --- |

Please confirm management actions have been carried out each shift with your initials, if actions not achieved please document a variance as “V” in the initial box. All variances must be recorded with explanation on page opposite.

| **POST-OP Management Plan:**  **Day of Surgery** |
| --- |

| **1&2) Nursing management following discharge to SHDU/ITU** | **Time of discharge from recovery ………** | | | |
| --- | --- | --- | --- | --- |
|  | **Initial for each shift.** | **am** | **pm** | **nocte** |
| Observations monitored and recorded hourly. | |  |  |  |
| Hourly fluid balance recorded on fluid chart. | |  |  |  |
| Urine output 0.33 of a ml per kg of body weight per hr over 4 hours. | |  |  |  |
| Wound dressings observed regularly for signs of bleeding or infection. | |  |  |  |
| Check chest drain is swinging and monitor for output.  1 x drain/ 2 x drains**.**  Record on fluid balance chart as per label on drain. | |  |  |  |
| Administer IV PPI as prescribed. | |  |  |  |
| Maintain nasogastric tube on free drainage | |  |  |  |
| Nurse on Hill-Rom bed. | |  |  |  |
| **3) PAIN** | | **am** | **pm** | **nocte** |
| EPIDURAL / PCA / Regional analgesia, observations as per protocol. | |  |  |  |
| If pain score 2-3 refer to On Call anaesthetist. **YES/N/A** | |  |  |  |
| IV Paracetamol given TDS as prescribed. | |  |  |  |
| Patient observed for signs of nausea or vomiting. | |  |  |  |
| **4) CHEST/MOBILITY** | | **am** | **pm** | **nocte** |
| Deep breathing exercises and cough encouraged. | |  |  |  |
| Patient sat out of bed for 2 hours. | |  |  |  |
| IPC pumps attached when in bed. | |  |  |  |
| **5) NUTRITIONAL AND FLUID INTAKE** | | **am** | **pm** | **nocte** |
| IV maintenance fluid as prescribed by anaesthetist. Then follow EROS policy  Dextrose Saline + 2g kcl 1ml/kg/hr | |  |  |  |
| Patient to remain NBM with pink sticks/mouth care offered regularly. **STEP 1** | |  |  |  |
| Administer sterile water at 10ml/hr via jejunostomy, from 10pm. Decant bottle and giving set must be changed 4 hourly in accordance with hospital policy. (See appendix) Refer to dietician via EQuest. **Complete: Yes/No** | |  |  |  |
| No oral medication to be given. Discuss with pharmacy/ prescriber alternative routes for necessary drugs. | |  |  |  |

Oesophagogastric Ward Round

| Nurse |  |
| --- | --- |
| Consultant | Byrne Kelly Underwood |
| Registrar |  |
| SHO |  |
| F1 |  |

Date / / Time

**Appearance**

**Update**

**O/E**

**Obs**  Apyrexial 

Stable 

**PMH**

EROS Yes  No 

**Output** NG

Chest Drain 1. 2. 3.

24° Urine output

Abdominal drain

**IV Fluids** Yes  No  Reason:

**Nutrition** Oral  Jej  TPN 

**Step**  1 2 3 4 5 6

**Antibiotics** No  Yes  - Day _____

VTE

Assessed 

Prescribed 

Catheter (reason):

Yes  No  Long-term 

**Significant blood results:**

**NUn score**

Social for discharge

Not required 

Section 2 signed 

Section 5 signed 

Rehab referred 

**Plan:**

Bloods today Yes  No 

Estimated Discharge Date:

**HMR** done Yes  No 

**Diagnosis**

**Procedure** **Date POD**

Signed: Name: Bleep: Designation

Therapy

Verbal consent obtained from patient 

| **NURSING DOCUMENTATION OF VARIANCE AND SUPPLEMENTARY NOTES.** | | |
| --- | --- | --- |
| *23/10/08*  *10.45* | ***(Example)***  *V(5) = Patient unable to drink second Fortisip as feeling nauseous. Cyclizine given IV. With good effect. (“5” relates to section of daily plan).* | *I. Fecher RN* |

| **EROS Care Pathway**  Day 1 Post Surgery (ITU/SHDU)  07.00am- 06.59am | (Patient Label) | | | | |
| --- | --- | --- | --- | --- | --- |
| **DATE:** |
| **PLEASE NOTE:** Please confirm management actions have been carried out each shift with your initials, if actions not achieved please document a variance as “V” in the initial box. All variances must be recorded with explanation on page opposite. | | | | | |
| **1) GENERAL OBSERVATIONS** | | **am** | **pm** | | **nocte** |
| Observations recorded as per post-op protocol. | |  |  | |  |
| Patient monitored for signs of nausea and vomiting. If present refer to postoperative N&V protocol. | |  |  | |  |
| Blood taken for FBC, U&E’s, Magnesium. Phosphate, CRP. | |  |  | |  |
| Administer IV PPI as prescribed. | |  |  | |  |
| Maintain nasogastric tube on free drainage | |  |  | |  |
| **2) FLUID BALANCE** | | **am** | **pm** | | **nocte** |
| Fluid balance chart maintained, urinary catheter measurements = 0.33ml/kg/hr. | |  |  | |  |
| Chart total chest drain output for each drain for last 24 hours at 7am and mark bottle/s. | |  |  | |  |
| Check chest drain is swinging and monitor for output.  1 x drain/ 2 x drains. | |  |  | |  |
| **3) PAIN** | | **am** | **pm** | | **nocte** |
| EPIDURAL / PCA/ Regional analgesia observations as per protocol. | |  |  | |  |
| If pain score 2-3 patient referred to Acute Pain Team. | | **Y/N** | **Y/N** | | **Y/N** |
| Administer IV Paracetamol TDS as prescribed. | |  |  | |  |
| Antiemetic prescribed PRN **Given:** | | **Y/N** | **Y/N** | | **Y/N** |
| **4) CHEST/MOBILITY** | | **am** | **pm** | | **nocte** |
| Deep breathing and coughing encouraged. | |  |  | |  |
| Patient sat out of bed for 4 hours total. | |  |  | |  |
| Monitored for signs of chest infection. | |  |  | |  |
| 2x Walks**(See p.8 for patients individual targets.)** | 1^st^ walk**25m / 50 m** | 2^nd^ walk 25m / 50 m | | | |
| IPC pumps attached when in bed. | |  |  | |  |
| **5) NUTRITION AND FLUID INTAKE** | | **am** | | **pm** | **nocte** |
| IV maintenance fluid as per EROS policy Dextrose Saline + 2g kcl 1ml/kg/hr | |  | |  |  |
| Patient to remain **NBM** (pink sticks/mouth care only) **STEP 1** | |  | |  |  |
| Administer jejunal feeding as per regimen. Either standard or refeeding risk regimen decided by dietician. (See appendix) Refer to dietician via EQuest. **Complete: Yes/No** | |  | |  |  |
| No oral medication to be given. Discuss with pharmacy/ prescriber alternative routes for necessary drugs. | |  | |  |  |

Oesophagogastric Ward Round

| Nurse |  |
| --- | --- |
| Consultant | Byrne Kelly Underwood |
| Registrar |  |
| SHO |  |
| F1 |  |

Date / / Time

**Appearance**

**Update**

**O/E**

**Obs**  Apyrexial 

Stable 

**PMH**

EROS Yes  No 

**Output** NG

Chest Drain 1. 2. 3.

24° Urine output

Abdominal drain

**IV Fluids** Yes  No  Reason:

**Nutrition** Oral  Jej  TPN 

**Step**  1 2 3 4 5 6

**Antibiotics** No  Yes  - Day _____

VTE

Assessed 

Prescribed 

Catheter (reason):

Yes  No  Long-term 

Social for discharge

Not required 

Section 2 signed 

Section 5 signed 

Rehab referred 

**Significant blood results:**

**NUn score**

**Plan:**

Bloods today Yes  No 

Estimated Discharge Date:

**HMR** done Yes  No 

Signed: Name: Bleep: Designation

Therapy

Verbal consent obtained from patient 

| **NURSING DOCUMENTATION OF VARIANCE AND SUPPLEMENTARY NOTES.** | | |
| --- | --- | --- |
| *23/10/08*  *10.45* | ***(Example)***  *V(5) = Patient unable to drink second Fortisip as feeling nauseous. Cyclizine given IV. With good effect. (“5” relates to section of daily plan).* | *I. Fecher RN* |

| **EROS Care Pathway**  Day 2 Post Surgery (ITU/SHDU)  07.00am- 06.59am | (Patient Label) |
| --- | --- |
| **DATE:** |
| **PLEASE NOTE:** Please confirm management actions have been carried out each shift with your initials, if actions not achieved please document a variance as “V” in the initial box. All variances must be recorded with explanation on page opposite. | |

| **1) GENERAL OBSERVATIONS** | **am** | **pm** | **nocte** |
| --- | --- | --- | --- |
| Observations recorded as per post-op protocol. |  |  |  |
| Patient monitored for signs of nausea and vomiting. If present refer to postoperative N&V protocol. |  |  |  |
| Administer IV PPI as prescribed. |  |  |  |
| Blood taken for FBC, U&E’s, Magnesium. Phosphate, CRP. |  |  |  |
| Consider nasogastric tube removal on consultant decision |  |  |  |

| **2) FLUID BALANCE** | | | | **am** | **pm** | **nocte** |
| --- | --- | --- | --- | --- | --- | --- |
| Fluid balance chart maintained, urinary catheter measurements = 0.33ml/kg/hr. | | | |  |  |  |
| Chart total chest drain output for each drain for last 24 hours at 7am and mark bottle/s. | | | |  |  |  |
| Check chest drain is swinging and monitor for output.  1 x drain/ 2 x drains. | | | |  |  |  |
| **3) PAIN** | | | **am** | | **pm** | **nocte** |
| EPIDURAL / PCA/ Regional analgesia observations as per protocol. | | |  | |  |  |
| If pain score persistently > 3 patient referred to Acute Pain Team. | | | **Y/N** | | **Y/N** | **Y/N** |
| Administer IV Paracetamol TDS as prescribed. | | |  | |  |  |
| Antiemetic prescribed PRN **Given:** | | | **Y/N** | | **Y/N** | **Y/N** |
| **4) CHEST/MOBILITY** | | | **am** | | **pm** | **nocte** |
| Deep breathing and coughing encouraged. | | |  | |  |  |
| Patient sat out of bed for 6 hours total. | | |  | |  |  |
| Monitored for signs of chest infection. | | |  | |  |  |
| 3x Walks**(See p.8 for patients individual targets.)** | 1^st^ walk **25m / 50 m** | 2^nd^ walk**25m / 50 m** | 3rd walk **25m / 50 m** | | | |
| IPC pumps attached when in bed. | | |  | |  |  |
| **5) NUTRITION AND FLUID INTAKE** | | | **am** | | **pm** | **nocte** |
| IV maintenance fluid as per EROS policy Dextrose Saline + 2g kcl 1ml/kg/hr | | |  | |  |  |
| Increase oral intake to 50ml/hr (water/black tea/coffee), **STEP 2** | | |  | |  |  |
| Administer jejunal feeding as per regimen. Either standard or refeeding risk regimen decided by dietician. (See appendix if no regimen from dietician) | | |  | |  |  |
| Give essential oral medication in liquid/soluble formulations only.  Ensure effervescent tablets have stopped fizzing prior to administration. | | |  | |  |  |

Oesophagogastric Ward Round

| Nurse |  |
| --- | --- |
| Consultant | Byrne Kelly Underwood |
| Registrar |  |
| SHO |  |
| F1 |  |

Date / / Time

**Appearance**

**Update**

**O/E**

**Obs**  Apyrexial 

Stable 

**PMH**

EROS Yes  No 

**Output** NG

Chest Drain 1. 2. 3.

24° Urine output

Abdominal drain

**IV Fluids** Yes  No  Reason:

**Nutrition** Oral  Jej  TPN 

**Step**  1 2 3 4 5 6

**Antibiotics** No  Yes  - Day _____

VTE

Assessed 

Prescribed 

Catheter (reason):

Yes  No  Long-term 

Social for discharge

Not required 

Section 2 signed 

Section 5 signed 

Rehab referred 

**Significant blood results:**

**NUn score**

**Plan:**

Bloods today Yes  No 

Estimated Discharge Date:

**HMR** done Yes  No 

Signed: Name: Bleep: Designation

Therapy

Verbal consent obtained from patient 

| **NURSING DOCUMENTATION OF VARIANCE AND SUPPLEMENTARY NOTES.** | | |
| --- | --- | --- |
| *23/10/08*  *10.45* | ***(Example)***  *V(5) = Patient unable to drink second Fortisip as feeling nauseous. Cyclizine given IV. With good effect. (“5” relates to section of daily plan).* | *I. Fecher RN* |

| **EROS Care Pathway**  Day 3 Post Surgery (ITU/SHDU/Ward F7)  07.00am- 06.59am | (Patient Label) |
| --- | --- |
| **DATE:** |
| **PLEASE NOTE:** Please confirm management actions have been carried out each shift with your initials, if actions not achieved please document a variance as “V” in the initial box. All variances must be recorded with explanation on page opposite. | |

| **1) GENERAL OBSERVATIONS** | **am** | **pm** | **nocte** |
| --- | --- | --- | --- |
| Observations recorded as per post-op protocol. Commence MEWS on ward. |  |  |  |
| Patient monitored for signs of nausea and vomiting. If present refer to postoperative N&V protocol. |  |  |  |
| Blood taken for FBC, U&E’s, Magnesium. Phosphate, CRP. |  |  |  |
| Consider nasogastric tube removal on consultant decision if not already done |  |  |  |
| Administer IV PPI as prescribed. |  |  |  |

| **2) FLUID BALANCE** | | | | **am** | **pm** | | **nocte** |
| --- | --- | --- | --- | --- | --- | --- | --- |
| Fluid balance chart maintained, urinary catheter measurements = 0.33ml/kg/hr. | | | |  |  | |  |
| Chart total chest drain output for each drain for last 24 hours at 7am and mark bottle/s. | | | |  |  | |  |
| **3) PAIN** | | | | **am** | **pm** | | **nocte** |
| EPIDURAL / PCA/ Regional analgesia observations as per protocol. | | | |  |  | |  |
| If pain score 2-3 patient referred to Acute Pain Team. | | | | **Y/N** | **Y/N** | | **Y/N** |
| Administer IV Paracetamol TDS as prescribed. | | | |  |  | |  |
| Antiemetic prescribed PRN **Given:** | | | | **Y/N** | **Y/N** | | **Y/N** |
| **4) CHEST/MOBILITY** | | | | **am** | **pm** | | **nocte** |
| Deep breathing and coughing encouraged. | | | |  |  | |  |
| Patient sat out of bed for 6 hours total. | | | |  |  | |  |
| Monitored for signs of chest infection, refer to physiotherapist if appropriate. | | | |  |  | |  |
| 4x Walks**(See p.8 for patient’s individual targets.)** | 1^st^ walk **25m / 50 m** | 2^nd^ walk**25m / 50 m** | 3rd walk **25m / 50 m** | | 4^th^ walk  **25 / 50 m** | | |
| **5) NUTRITION AND FLUID INTAKE** | | | | **am** | | **pm** | **nocte** |
| IV maintenance fluid as per EROS policy Dextrose Saline + 2g kcl 1ml/kg/hr | | | |  | |  |  |
| Continue oral intake at 50ml/hr (water/black tea/coffee) **STEP 2** | | | |  | |  |  |
| Administer jejunal feeding as per regimen. Either standard or refeeding risk regimen decided by dietician. | | | |  | |  |  |
| Give essential oral medication in liquid/soluble formulations only.  Ensure effervescent tablets have stopped fizzing prior to administration. | | | |  | |  |  |

| **6 DISCHARGE PLANNING** | **am** | **pm** | **nocte** |
| --- | --- | --- | --- |
| **Plan of care discussed with patient.** |  |  |  |

Oesophagogastric Ward Round

| Nurse |  |
| --- | --- |
| Consultant | Byrne Kelly Underwood |
| Registrar |  |
| SHO |  |
| F1 |  |

Date / / Time

**Appearance**

**Update**

**O/E**

**Obs**  Apyrexial 

Stable 

**PMH**

**IV Fluids** Yes  No  Reason:

**Nutrition** Oral  Jej  TPN 

**Step**  1 2 3 4 5 6

**Antibiotics** No  Yes  - Day _____

VTE

Assessed 

Prescribed 

**Significant blood results:**

**NUn score**

Catheter (reason):

Yes  No  Long-term 

Social for discharge

Not required 

Section 2 signed 

Section 5 signed 

Rehab referred 

**Plan:**

Bloods today Yes  No 

Estimated Discharge Date:

**HMR** done Yes  No 

EROS Yes  No 

Signed: Name: Bleep: Designation

**Output** NG

Chest Drain 1. 2. 3.

24° Urine output

Abdominal drain

Therapy

Verbal consent obtained from patient 

| **NURSING DOCUMENTATION OF VARIANCE AND SUPPLEMENTARY NOTES.** | | |
| --- | --- | --- |
| *23/10/08*  *10.45* | ***(Example)***  *V(5) = Patient unable to drink second Fortisip as feeling nauseous. Cyclizine given IV. With good effect. (“5” relates to section of daily plan).* | *I. Fecher RN* |

| EROS Care Pathway  Day 4 Post Surgery (Monitored Bed Ward E5)  07.00am- 06.59am | (Patient Label) |
| --- | --- |
| **DATE:** |
| **PLEASE NOTE:** Please confirm management actions have been carried out each shift with your initials, if actions not achieved please document a variance as “V” in the initial box. All variances must be recorded with explanation on page opposite. | |

| **1) GENERAL OBSERVATIONS** | **am** | **pm** | **nocte** |
| --- | --- | --- | --- |
| Observations and MEWS recorded as per protocol. |  |  |  |
| Patient monitored for signs of nausea and vomiting. If present refer to postoperative N&V protocol. |  |  |  |
| Blood taken for FBC, U&E’s, Magnesium. Phosphate, CRP and Albumin (required for NUn scure calculation) |  |  |  |
| Administer IV PPI as prescribed. |  |  |  |

| **2) FLUID BALANCE** | **am** | **pm** | **nocte** |
| --- | --- | --- | --- |
| Fluid balance chart maintained, urinary output = 0.33ml/kg/hr. |  |  |  |
| Chart total chest drain output for each drain for last 24 hours at 7am and mark bottle/s. |  |  |  |
| If chest drain output <150ml in 24 hours and output clear consider removal.  (check documentation in medical notes). |  |  |  |
| Chest Xray performed and reviewed within 1 hour of chest drain removal. |  |  |  |
| TWOC at midnight unless already removed. (check documentation in medical notes). |  |  |  |

| **3) PAIN** | **am** | | **pm** | **nocte** |
| --- | --- | --- | --- | --- |
| EPIDURAL / Regional analgesia observations as per protocol. Discontinued as Day 4 **Y / N** | |  |  |  |
| PCA continued | | **Y/N** | **Y/N** | **Y/N** |
| If pain score persistently >3 patient referred to Acute Pain Team. | | **Y/N** | **Y/N** | **Y/N** |
| Administer Paracetamol orally QDS as prescribed. | |  |  |  |
| Administer liquid ibuprofen via jejunostomy as prescribed. | |  |  |  |
| Antiemetic prescribed PRN **Given:** | | **Y/N** | **Y/N** | **Y/N** |

| **4) CHEST/MOBILITY** | | | | | **am** | | **pm** | **nocte** |
| --- | --- | --- | --- | --- | --- | --- | --- | --- |
| Deep breathing and coughing encouraged. | | | | |  | |  |  |
| Patient sat out of bed for 6 hours total. | | | | |  | |  |  |
| 5 x Walks **(See p.8 for patient’s individual targets.)** | 1^st^ walk **10m / 50m / 100m** | 2^nd^ walk **10m / 50m / 100m** | 3^rd^ walk **10m / 50m / 100m** | 4^th^ walk **10m / 50m / 100m** | | 5^th^ walk **10m / 50m / 100m** | | |

| **5) NUTRITION AND FLUID INTAKE** | **am** | **pm** | **nocte** |
| --- | --- | --- | --- |
| IV maintenance fluid as per EROS policy Dextrose Saline + 2g kcl 1ml/kg/hr. Discontinue when tolerating >1ml/kg/hr of combined oral and jejunal intake. |  |  |  |
| Administer jejunal feeding as per regimen. Either standard or refeeding risk regimen decided by dietician. |  |  |  |
| Give essential oral medication given in liquid/soluble formulations only.  Ensure effervescent tablets have stopped fizzing prior to administration. |  |  |  |

| **PATIENT WILL FOLLOW 5a) OR 5b) DEPENDING UPON RESULTS OF NUn SCORE:** | | | | | | |
| --- | --- | --- | --- | --- | --- | --- |
| **5a)** | | | **am** | | **pm** | **nocte** |
| Increase oral intake to free fluids. **STEP 3** | | |  | |  |  |
| 3 x Fortisip/juice | **Mid am** | **Mid pm** | | **Evening** | | |
| Oral medication can be given. Tablets to be crushed and effervescent tablets must not be fizzing. | | |  | |  |  |

| **5b)** | **am** | **pm** | **nocte** |
| --- | --- | --- | --- |
| Continue oral intake at 50ml/hr (water/black tea/coffee) STEP 2 |  |  |  |
| Give essential oral medication given in liquid/soluble formulations only.  Ensure effervescent tablets have stopped fizzing prior to administration. |  |  |  |

| **6) HOME JEJUNAL TUBE EDUCATION** | **am** | **pm** | **nocte** |
| --- | --- | --- | --- |
| Identify who will administer home jej flush or feeding. **PATIENT/CARER** |  |  |  |
| Liaise with dietician to order home equipment and/ or sterile water |  |  |  |
| Patient/carer education commenced. |  |  |  |

| **7) STEP DOWN FROM CRITICAL CARE** | **am** | **pm** | **nocte** |
| --- | --- | --- | --- |
| Patient fit for discharge from critical care.  Step down to ward E5  If no, reason …………………………………………… | Yes/No  Yes/No |  |  |

Oesophagogastric Ward Round

**Appearance**

**Update**

**O/E**

**Obs**  Apyrexial 

Stable 

**PMH**

**IV Fluids** Yes  No  Reason:

**Nutrition** Oral  Jej  TPN 

**Step**  1 2 3 4 5 6

**Antibiotics** No  Yes  - Day _____

VTE

Assessed 

Prescribed 

**Significant blood results:**

**NUn score**

Catheter (reason):

Yes  No  Long-term 

Social for discharge

Not required 

Section 2 signed 

Section 5 signed 

Rehab referred 

**Plan:**

Bloods today Yes  No 

Estimated Discharge Date:

**HMR** done Yes  No 

EROS Yes  No 

Signed: Name: Bleep: Designation

| Nurse |  |
| --- | --- |
| Consultant | Byrne Kelly Underwood |
| Registrar |  |
| SHO |  |
| F1 |  |

Date / / Time

**Output** NG

Chest Drain 1. 2. 3.

24° Urine output

Abdominal drain

Therapy

Verbal consent obtained from patient 

| **NURSING DOCUMENTATION OF VARIANCE AND SUPPLEMENTARY NOTES.** | | |
| --- | --- | --- |
| *23/10/08*  *10.45* | ***(Example)***  *V(5) = Patient unable to drink second Fortisip as feeling nauseous. Cyclizine given IV. With good effect. (“5” relates to section of daily plan).* | *I. Fecher RN* |

| EROS Care Pathway  Day 5 Post Surgery  Ward E5  07.00am- 06.59am | (Patient Label) |
| --- | --- |
| **DATE:** |
| **PLEASE NOTE:** Please confirm management actions have been carried out each shift with your initials, if actions not achieved please document a variance as “V” in the initial box. All variances must be recorded with explanation on page opposite. | |

| **1) GENERAL OBSERVATIONS** | **am** | **pm** | **nocte** |
| --- | --- | --- | --- |
| Observations and MEWS recorded as per protocol. |  |  |  |
| Patient monitored for signs of nausea and vomiting. If present refer to postoperative N&V protocol. |  |  |  |
| Administer PO PPI as prescribed. |  |  |  |

| **2) FLUID BALANCE** | **am** | **pm** | **nocte** |
| --- | --- | --- | --- |
| Fluid balance chart maintained, urinary output = 0.33ml/kg/hr. |  |  |  |
| Chart total chest drain output for each drain for last 24 hours at 7am and mark bottle/s. |  |  |  |
| If chest drain output <150ml in 24 hours and output clear consider removal.  (check documentation in medical notes.) |  |  |  |
| Chest X-ray performed and reviewed within 1 hour of chest drain removal. |  |  |  |
| TWOC at midnight unless already removed. |  |  |  |

| **3) PAIN** | **am** | **pm** | | **nocte** |
| --- | --- | --- | --- | --- |
| EPIDURAL / Regional analgesia discontinued as Day 5 | **Y / N** | | | |
| PCA stopped. | **Y/N** | | **Y/N** | **Y/N** |
| If pain score >3 patient referred to Acute Pain Team. | **Y/N** | | **Y/N** | **Y/N** |
| Administer Paracetamol orally QDS as prescribed. |  | |  |  |
| Administer liquid ibuprofen via jejunostomy as prescribed/if required. |  | |  |  |
| Antiemetic prescribed PRN **Given:** | **Y/N** | | **Y/N** | **Y/N** |

| **4) CHEST/MOBILITY** | | | | | **am** | | **pm** | | **nocte** |
| --- | --- | --- | --- | --- | --- | --- | --- | --- | --- |
| Deep breathing and coughing encouraged. | | | | |  | |  | |  |
| Patient sat out of bed for 6 hours total. | | | | |  | |  | |  |
| 6 x Walks **(See p.8 for patient’s individual targets.)** | 1^st^ walk 10m / 50m / 100m | 2^nd^ walk 10m / 50m / 100m | 3^rd^ walk 10m / 50m / 100m | 4^th^ walk 10m / 50m / 100m | | 5^th^ walk 10m / 50m / 100m | | 6^th^ walk 10m / 50m / 100m | |

| **5) NUTRITION AND FLUID INTAKE** | **am** | **pm** | **nocte** |
| --- | --- | --- | --- |
| IV maintenance fluid as per EROS policy Dextrose Saline + 2g kcl 1ml/kg/hr. Discontinue when tolerating >1ml/kg/hr of combined oral and jejunal intake. |  |  |  |
| Administer jejunal feeding as per regimen. Either standard or refeeding risk regimen decided by dietician. |  |  |  |
| Give essential oral medication given in liquid/soluble formulations only.  Ensure effervescent tablets have stopped fizzing prior to administration. |  |  |  |

| **PATIENT WILL FOLLOW 5a) OR 5b) DEPENDING UPON RESULTS OF NUn SCORE.** | | | | | | |
| --- | --- | --- | --- | --- | --- | --- |
| **5a)** | | | **am** | | **pm** | **nocte** |
| Continue oral intake at free fluids. STEP 3 | | |  | |  |  |
| 3 x Fortisip/juice | **Mid am** | **Mid pm** | | **Evening** | | |
| Oral medication can be given. Tablets to be crushed and effervescent tablets must not be fizzing. | | |  | |  |  |

| **5b)** | **am** | **pm** | **nocte** |
| --- | --- | --- | --- |
| Continue oral intake at 50ml/hr (water/black tea/coffee) STEP 2 |  |  |  |
| Give essential oral medication given in liquid/soluble formulations only.  Ensure effervescent tablets have stopped fizzing prior to administration. |  |  |  |

| **6) HOME JEJUNAL TUBE EDUCATION** | **am** | **pm** | **nocte** |
| --- | --- | --- | --- |
| Identify who will administer home jej flush or feeding. **PATIENT/CARER** |  |  |  |
| Liaise with dietician to order home equipment and/or sterile water |  |  |  |
| Patient/carer education continues. |  |  |  |

| **7) DISCHARGE PLANNING** | **am** | **pm** | **nocte** |
| --- | --- | --- | --- |
| **Plan of care discussed with patient.** |  |  |  |
| **Patient fit for discharge in 3 days? YES/NO (If yes complete discharge planning checklist)**  **If “NO” reason .....................................................................................................................................** | | | |

Oesophagogastric Ward Round

| Nurse |  |
| --- | --- |
| Consultant | Byrne Kelly Underwood |
| Registrar |  |
| SHO |  |
| F1 |  |

Date / / Time

**Appearance**

**Update**

**O/E**

**Obs**  Apyrexial 

Stable 

**PMH**

Estimated Discharge Date:

EROS Yes  No 

**Output** NG

Chest Drain 1. 2. 3.

24° Urine output

Abdominal drain

**IV Fluids** Yes  No  Reason:

**Nutrition** Oral  Jej  TPN 

**Step**  1 2 3 4 5 6

**Antibiotics** No  Yes  - Day _____

VTE

Assessed 

Prescribed 

Catheter (reason):

Yes  No  Long-term 

Social for discharge

Not required 

Section 2 signed 

Section 5 signed 

Rehab referred 

**Significant blood results:**

**NUn score**

**Plan:**

Bloods today Yes  No 

**HMR** done Yes  No 

Signed: Name: Bleep: Designation

Therapy

Verbal consent obtained from patient 

| **NURSING DOCUMENTATION OF VARIANCE AND SUPPLEMENTARY NOTES.** | | |
| --- | --- | --- |
| *23/10/08*  *10.45* | ***(Example)***  *V(5) = Patient unable to drink second Fortisip as feeling nauseous. Cyclizine given IV. With good effect. (“5” relates to section of daily plan).* | *I. Fecher RN* |

| EROS Care Pathway  Day 6 Post Surgery  Ward E5  07.00am- 06.59am | (Patient Label) | | | |
| --- | --- | --- | --- | --- |
| **DATE:** |
| **PLEASE NOTE:** Please confirm management actions have been carried out each shift with your initials, if actions not achieved please document a variance as “V” in the initial box. All variances must be recorded with explanation on page opposite. | | | | |
| **1) GENERAL OBSERVATIONS** | | **am** | **pm** | **nocte** |
| Observations and MEWS recorded as per protocol. | |  |  |  |
| Patient monitored for signs of nausea and vomiting. If present refer to postoperative N&V protocol. | |  |  |  |
| Administer PO PPI as prescribed. | |  |  |  |
| **2) FLUID BALANCE** | | **am** | **pm** | **nocte** |
| Fluid balance chart maintained, urinary output = 0.33ml/kg/hr. | |  |  |  |
| Chart total chest drain output for each drain for last 24 hours at 7am and mark bottle/s. | |  |  |  |
| If chest drain output <150ml in 24 hours and output clear consider removal.  (check documentation in medical notes). | |  |  |  |
| Chest Xray performed and reviewed within 1 hour of chest drain removal. | |  |  |  |
| If chest drain still draining obtain long term plan from consultant. | | **Y / N** |  | |

| **3) PAIN** | **am** | **pm** | **nocte** |
| --- | --- | --- | --- |
| Pain managed with oral/enterally administered analgesia | **Y/N** | **Y/N** | **Y/N** |
| If pain score 2-3 patient referred to Acute Pain Team. | **Y/N** | **Y/N** | **Y/N** |
| Antiemetic prescribed PRN **Given:** | **Y/N** | **Y/N** | **Y/N** |
| Administer liquid ibuprofen via jejunostomy as prescribed. |  |  |  |

| **4) CHEST/MOBILITY** | | | | | **am** | | **pm** | | **nocte** |
| --- | --- | --- | --- | --- | --- | --- | --- | --- | --- |
| Deep breathing and coughing encouraged. | | | | |  | |  | |  |
| Patient sat out of bed for 6 hours total. | | | | |  | |  | |  |
| 6 x Walks **(See p.8 for patient’s individual targets.)** | 1^st^ walk 10m / 50m / 100m | 2^nd^ walk 10m / 50m / 100m | 3^rd^ walk 10m / 50m / 100m | 4^th^ walk 10m / 50m / 100m | | 5^th^ walk 10m / 50m / 100m | | 6^th^ walk 10m / 50m / 100m | |

| **5 NUTRITION AND FLUID INTAKE** | **am** | **pm** | | **nocte** | |
| --- | --- | --- | --- | --- | --- |
| IV maintenance discontinued as combined oral/jejunal intake >1ml/kg/hr. | **Y / N** | | | | |
| Administer jejunal feeding as per regimen. Either standard or refeeding risk regimen decided by dietician. |  | |  | |  |

| **PATIENT WILL FOLLOW 5a) OR 5b) DEPENDING UPON RESULTS OF NUn SCORE** | | | | | | |
| --- | --- | --- | --- | --- | --- | --- |
| **5a)** | | | **am** | | **pm** | **nocte** |
| Increase oral intake to Puree C Diet. STEP 4 | | |  | |  |  |
| 3 x Fortisip/juice | **Mid am** | **Mid pm** | | **Evening** | | |
| Oral medication can be given. Tablets to be crushed and effervescent tablets must not be fizzing. | | |  | |  |  |

| **5b)** | **am** | **pm** | **nocte** |
| --- | --- | --- | --- |
| Continue oral intake at 50ml/hr (water/black tea/coffee) **STEP 2** | **Y/N** | **Y/N** | **Y/N** |
| Give essential oral medication given in liquid/soluble formulations only.  Ensure effervescent tablets have stopped fizzing prior to administration. | **Y/N** | **Y/N** | **Y/N** |

| **6) HOME JEJUNAL TUBE EDUCATION** | **am** | **pm** | **nocte** |
| --- | --- | --- | --- |
| Identify who will administer home jej tube flush or feeding. **PATIENT/CARER** |  |  |  |
| Liaise with dietician to order home equipment and/or sterile water |  |  |  |
| Patient/carer education continues. Patient/carer supported with feed administration. |  |  |  |

| **7) DISCHARGE PLANNING** | **am** | **pm** | **nocte** |
| --- | --- | --- | --- |
| **Plan of care discussed with patient.** |  |  |  |
| **Patient fit for discharge in 2 days? YES/NO (If yes complete discharge planning checklist)**  **If “NO” reason .....................................................................................................................................** | | | |

Oesophagogastric Ward Round

| Nurse |  |
| --- | --- |
| Consultant | Byrne Kelly Underwood |
| Registrar |  |
| SHO |  |
| F1 |  |

Date / / Time

**Appearance**

**Update**

**O/E**

**Obs**  Apyrexial 

Stable 

**PMH**

**IV Fluids** Yes  No  Reason:

**Nutrition** Oral  Jej  TPN 

**Step**  1 2 3 4 5 6

**Antibiotics** No  Yes  - Day _____

VTE

Assessed 

Prescribed 

**Significant blood results:**

**NUn score**

Catheter (reason):

Yes  No  Long-term 

Social for discharge

Not required 

Section 2 signed 

Section 5 signed 

Rehab referred 

**Plan:**

Bloods today Yes  No 

Estimated Discharge Date:

**HMR** done Yes  No 

EROS Yes  No 

Signed: Name: Bleep: Designation

**Output** NG

Chest Drain 1. 2. 3.

24° Urine output

Abdominal drain

Therapy

Verbal consent obtained from patient 

| **NURSING DOCUMENTATION OF VARIANCE AND SUPPLEMENTARY NOTES.** | | |
| --- | --- | --- |
| *23/10/08*  *10.45* | ***(Example)***  *V(5) = Patient unable to drink second Fortisip as feeling nauseous. Cyclizine given IV. With good effect. (“5” relates to section of daily plan).* | *I. Fecher RN* |

| EROS Care Pathway  Day 7 Post Surgery  Ward E5  07.00am- 06.59am | (Patient Label) |
| --- | --- |
| **DATE:** |
| **PLEASE NOTE:** Please confirm management actions have been carried out each shift with your initials, if actions not achieved please document a variance as “V” in the initial box. All variances must be recorded with explanation on page opposite. | |

| **1) GENERAL OBSERVATIONS** | **am** | **pm** | **nocte** |
| --- | --- | --- | --- |
| Observations and MEWS recorded as per protocol. |  |  |  |
| Patient monitored for signs of nausea and vomiting. If present refer to postoperative N&V protocol. |  |  |  |
| Administer PO PPI as prescribed. |  |  |  |

| **2) FLUID BALANCE** | **am** | **pm** | **nocte** |
| --- | --- | --- | --- |
| Fluid balance chart discontinued. |  |  |  |
| If chest drain still draining obtain long term plan from consultant. | **Y / N** | | |

| **3) PAIN** | **am** | **pm** | **nocte** |
| --- | --- | --- | --- |
| Pain managed with oral/enterally administered analgesia | **Y/N** | **Y/N** | **Y/N** |
| If pain score 2-3 patient referred to Acute Pain Team. | **Y/N** | **Y/N** | **Y/N** |
| Antiemetic prescribed PRN **Given:** | **Y/N** | **Y/N** | **Y/N** |
| Administer liquid ibuprofen via jejunostomy as prescribed. |  |  |  |

| **4) CHEST/MOBILITY** | | | | | **am** | | **pm** | | **nocte** |
| --- | --- | --- | --- | --- | --- | --- | --- | --- | --- |
| Deep breathing and coughing encouraged. | | | | |  | |  | |  |
| Patient sat out of bed for 6 hours total. | | | | |  | |  | |  |
| 6 x Walks **(See p.8 for patient’s individual targets.)** | 1^st^ walk 10m / 50m / 100m | 2^nd^ walk 10m / 50m / 100m | 3^rd^ walk 10m / 50m / 100m | 4^th^ walk 10m / 50m / 100m | | 5^th^ walk 10m / 50m / 100m | | 6^th^ walk 10m / 50m / 100m | |

| **5) NUTRITION AND FLUID INTAKE** | **am** | **pm** | | **nocte** | |
| --- | --- | --- | --- | --- | --- |
| IV maintenance discontinued as combined oral/jejunal intake >1ml/kg/hr. | **Y / N** | | | | |
| Administer jejunal feeding as per regimen. Either standard or refeeding risk regimen decided by dietician. |  | |  | |  |

| **PATIENT WILL FOLLOW 5a) OR 5b) DEPENDING UPON RESULTS OF NUn SCORE.** | | | | | | |
| --- | --- | --- | --- | --- | --- | --- |
| **5a)** | | | **am** | | **pm** | **nocte** |
| Continue oral intake as Puree C Diet. **STEP 4** | | |  | |  |  |
| 3 x Fortisip/juice | **Mid am** | **Mid pm** | | **Evening** | | |
| Oral medication can be given. Tablets to be crushed and effervescent tablets must not be fizzing. | | |  | |  |  |

| **5b)** | **am** | **pm** | **nocte** |
| --- | --- | --- | --- |
| Continue oral intake at 50ml/hr (water/black tea/coffee) **STEP 2** |  |  |  |
| Give essential oral medication given in liquid/soluble formulations only.  Ensure effervescent tablets have stopped fizzing prior to administration. |  |  |  |

| **6) HOME JEJUNAL TUBE EDUCATION** | **am** | **pm** | **nocte** |
| --- | --- | --- | --- |
| Patient/carer independent with feed and/or jejuna l flush administration. | **Y / N** | | |
| Feeding pump and feed supplies and/or sterile water available for patient at home | **Y / N N/a** | | |
| Giving sets, syringes and sterile water supplies available for patient to take home. | **Y / N** | | |

| **7) DISCHARGE PLANNING** | **am** | **pm** | **nocte** |
| --- | --- | --- | --- |
| **Plan of care discussed with patient.** |  |  |  |
| **Patient fit for discharge tomorrow? YES/NO (If yes complete discharge planning checklist)**  **If “NO” reason .....................................................................................................................................** | | | |
| Discharge summary drafted on eDocs | **Y/N** | |  |
| District nurse/Practice nurse form commenced | **Y/N** | |  |

Oesophagogastric Ward Round

| Nurse |  |
| --- | --- |
| Consultant | Byrne Kelly Underwood |
| Registrar |  |
| SHO |  |
| F1 |  |

Date / / Time

**Appearance**

**Update**

**O/E**

**Obs**  Apyrexial 

Stable 

**PMH**

EROS Yes  No 

**Output** NG

Chest Drain 1. 2. 3.

24° Urine output

Abdominal drain

**IV Fluids** Yes  No  Reason:

**Nutrition** Oral  Jej  TPN 

**Step**  1 2 3 4 5 6

**Antibiotics** No  Yes  - Day _____

VTE

Assessed 

Prescribed 

Catheter (reason):

Yes  No  Long-term 

Social for discharge

Not required 

Section 2 signed 

Section 5 signed 

Rehab referred 

**Significant blood results:**

**NUn score**

**Plan:**

Bloods today Yes  No 

Estimated Discharge Date:

**HMR** done Yes  No 

Signed: Name: Bleep: Designation

Therapy

Verbal consent obtained from patient 

| **NURSING DOCUMENTATION OF VARIANCE AND SUPPLEMENTARY NOTES.** | | |
| --- | --- | --- |
| *23/10/08*  *10.45* | ***(Example)***  *V(5) = Patient unable to drink second Fortisip as feeling nauseous. Cyclizine given IV. With good effect. (“5” relates to section of daily plan).* | *I. Fecher RN* |

| EROS Care Pathway  Discharge Day 8  07.00am- 06.59am | | (Patient Label) | |
| --- | --- | --- | --- |
| DATE: | |  |  |
| Please document all deviations from protocol opposite in Nursing notes. | | | |
| **DISCHARGE CHECKLIST Initial to confirm** | | | YES |
| Patient fit for discharge (if not fit then continue inpatient pathway day 8). | | |  |
| Patient independently mobile. | | |  |
| Patient willing to go home. | | |  |
| Patient discharge information booklet given and discussed. | | |  |
| Patient aware of signs and symptoms of potential post operative complications and knows whom to contact if concerned. Contact numbers provided. | | |  |
| **NUTRITION** | **General**  Advice given on future requirements  Seen by dietician  **Home Jejunal feeding and/or water flush**  Patient/carer able to administer feed and water flushes.  Patient has pump at home.  Ward to supply 7 days of syringes, sterile water and giving sets.  Dietician to obtain supply of feed. | |  |
| **WOUND CARE** | Practice/ District Nurse referral made if required. N.B. Home Jejunal feeding patients will require DN referral to obtain further equipment supplies at home**.** | |  |
|  | Dressings and staple removers supplied if required. | |  |
| **MEDICATIONS** | HMR written. | | . |
|  | TTO’s supplied, patient understands medication regime. | |  |
|  | POD’s returned to patient as per TTO. | |  |
| **DOCUMENTATION** | Letter to GP given | |  |
|  | Out patients appointment made for 2 weeks. | |  |
| Contact Number for patient 24hrs post discharge. (Include area code)EROS phone: 07768 447611 Ward E5: 02381204641 | | |  |
| **24 HOURS POST DISCHARGE** | | | |
| Follow up phone call made to patient by EROS Team.  Team member: Rachael Hole Date/Time: | | | |
| **2 WEEKS POST DISCHARGE** | | | |
| Follow up phone call made to patient. Discharged from EROS programme.  Team member: Rachael Hole Date / time: | | | |

| EROS Care Pathway  Day 8 INPATIENT  Post Surgery  Ward E5  07.00am- 06.59am | (Patient Label) |
| --- | --- |
| **DATE:** |
| **PLEASE NOTE:** Please confirm management actions have been carried out each shift with your initials, if actions not achieved please document a variance as “V” in the initial box. All variances must be recorded with explanation on page opposite. | |

| **1) GENERAL OBSERVATIONS** | **am** | **pm** | **nocte** |
| --- | --- | --- | --- |
| Observations and MEWS recorded as per protocol. |  |  |  |
| Patient monitored for signs of nausea and vomiting. If present refer to postoperative N&V protocol. |  |  |  |
| Administer PPI orally as prescribed. |  |  |  |

| **2) FLUID BALANCE** | **am** | **pm** | **nocte** |
| --- | --- | --- | --- |
| Fluid balance chart discontinued. |  |  |  |
| If chest drain still draining obtain long term plan from consultant. | **Y / N** | | |

| **3) PAIN** | **am** | **pm** | **nocte** |
| --- | --- | --- | --- |
| Pain managed with oral/enterally administered analgesia | **Y/N** | **Y/N** | **Y/N** |
| If pain score 2-3 patient referred to Acute Pain Team. | **Y/N** | **Y/N** | **Y/N** |
| Antiemetic prescribed PRN **Given:** | **Y/N** | **Y/N** | **Y/N** |
| Administer liquid ibuprofen via jejunostomy as prescribed. |  |  |  |

| **4) CHEST/MOBILITY** | | | | | **am** | | **pm** | | **nocte** |
| --- | --- | --- | --- | --- | --- | --- | --- | --- | --- |
| Deep breathing and coughing encouraged. | | | | |  | |  | |  |
| Patient sat out of bed for 6 hours total. | | | | |  | |  | |  |
| 6 x Walks **(See p.8 for patient’s individual targets.)** | 1^st^ walk 10m / 50m / 100m | 2^nd^ walk 10m / 50m / 100m | 3^rd^ walk 10m / 50m / 100m | 4^th^ walk 10m / 50m / 100m | | 5^th^ walk 10m / 50m / 100m | | 6^th^ walk 10m / 50m / 100m | |

| **5) NUTRITION AND FLUID INTAKE** | **am** | **pm** | | **nocte** | |
| --- | --- | --- | --- | --- | --- |
| IV maintenance discontinued as combined oral/jejunal intake >1ml/kg/hr. | **Y / N** | | | | |
| Administer jejunal feeding as per regimen. Either standard or refeeding risk regimen decided by dietician. |  | |  | |  |

| PATIENT WILL FOLLOW 5a) OR 5b) DEPENDING UPON RESULTS OF NUn SCORE. | | | | | | |
| --- | --- | --- | --- | --- | --- | --- |
| **5a)** | | | **am** | | **pm** | **nocte** |
| Continue oral intake as Puree C Diet. **STEP 4** | | |  | |  |  |
| 3 x Fortisip/juice | **Mid am** | **Mid pm** | | **Evening** | | |
| Oral medication can be given. Tablets to be crushed and effervescent tablets must not be fizzing. | | |  | |  |  |

| **5b)** | **am** | **pm** | **nocte** |
| --- | --- | --- | --- |
| Continue oral intake at 50ml/hr (water/black tea/coffee) **STEP 2** |  |  |  |
| Give essential oral medication given in liquid/soluble formulations only.  Ensure effervescent tablets have stopped fizzing prior to administration. |  |  |  |

| **6) HOME JEJUNAL TUBE EDUCATION** | **am** | **pm** | **nocte** |
| --- | --- | --- | --- |
| Patient/carer independent with feed and/or sterile water administration. | **Y / N** | | |
| Feeding pump and feed supplies available for patient at home | **Y / N/ N/a** | | |
| Giving sets, syringes and sterile water supplies available for patient to take home. | **Y / N** | | |

| **7) DISCHARGE PLANNING** | **am** | **pm** | **nocte** |
| --- | --- | --- | --- |
| **Plan of care discussed with patient.** |  |  |  |
| **Patient fit for discharge tomorrow? YES/NO (If yes complete discharge planning checklist)**  **If “NO” reason .....................................................................................................................................** | | | |
| Discharge summary drafted on eDocs | **Y/N** | |  |
| District nurse/ Practice nurse form commenced | **Y/N** | |  |

Oesophagogastric Ward Round

| Nurse |  |
| --- | --- |
| Consultant | Byrne Kelly Underwood |
| Registrar |  |
| SHO |  |
| F1 |  |

Date / / Time

**Appearance**

**Update**

**O/E**

**Obs**  Apyrexial 

Stable 

**PMH**

**Output** NG

Chest Drain 1. 2. 3.

24° Urine output

Abdominal drain

**IV Fluids** Yes  No  Reason:

**Nutrition** Oral  Jej  TPN 

**Step**  1 2 3 4 5 6

**Antibiotics** No  Yes  - Day _____

VTE

Assessed 

Prescribed 

**Significant blood results:**

**NUn score**

Catheter (reason):

Yes  No  Long-term 

Social for discharge

Not required 

Section 2 signed 

Section 5 signed 

Rehab referred 

**Plan:**

Bloods today Yes  No 

Estimated Discharge Date:

**HMR** done Yes  No 

EROS Yes  No 

Signed: Name: Bleep: Designation

Therapy

Verbal consent obtained from patient 

| **NURSING DOCUMENTATION OF VARIANCE AND SUPPLEMENTARY NOTES.** | | |
| --- | --- | --- |
| *23/10/08*  *10.45* | ***(Example)***  *V(5) = Patient unable to drink second Fortisip as feeling nauseous. Cyclizine given IV. With good effect. (“5” relates to section of daily plan).* | *I. Fecher RN* |

**C**ontinuation of Nursing Notes

| **Date/Time** | **Documentation** | **Signature/Designation** |
| --- | --- | --- |
|  |  |  |
|  |  |  |
|  |  |  |
|  |  |  |
|  |  |  |
|  |  |  |
|  |  |  |
|  |  |  |
|  |  |  |
|  |  |  |
|  |  |  |
|  |  |  |
|  |  |  |
|  |  |  |
|  |  |  |
|  |  |  |
|  |  |  |
|  |  |  |
|  |  |  |
|  |  |  |
|  |  |  |
|  |  |  |
|  |  |  |
|  |  |  |
|  |  |  |
|  |  |  |
|  |  |  |
|  |  |  |
|  |  |  |
|  |  |  |
|  |  |  |
|  |  |  |
|  |  |  |
|  |  |  |
|  |  |  |
|  |  |  |
|  |  |  |
|  |  |  |
|  |  |  |
|  |  |  |
