# Supplementary material for: The Development, Application and Analysis of an Enhanced Recovery Programme for Major Oesophagogastric Resection
Source: J Gastrointest Surg. 2017 Jan 24;21(4):614–21. doi: 10.1007/s11605-017-3363-8 (PMC5359364; doi:10.1007/s11605-017-3363-8)
Supplement: Supplementary file 4 — (DOC 174 kb) [file 11605_2017_3363_MOESM4_ESM.doc]

**Enhanced Recovery after Oesophagogastric Surgery (EROS)**

| **Guidelines** |  |
| --- | --- |
| **1** | Surgical out patient clinic |
| **2** | Pre-admission counselling |
| **3** | Pre-operative Nutrition |
| **4** | Pre-medication |
| **5** | Anaesthetic |
| **6** | Analgesia |
| **7** | Incision |
| **8** | Nasogastric tubes |
| **9** | Fluid management |
| **10** | Blood tests |
| **11** | Chest Drains |
| **12** | Nutrition |
| **13** | Bladder Catheters |
| **14** | Enhanced mobilisation |
| **15** | Discharge criteria |
| **16** | Post-discharge Follow-up |
| **17** | Re-admission criteria |
| **18** | Audit |

**1.**

| **Surgical Out Patients** |
| --- |
| - Counselling of patient and carers regarding proposed surgery - EROS pathway explained (patient, relatives and carers) - Patient information booklet provided - Baseline nutritional assessment to define patients who may require supplemental feeding pre- and/or post-operatively– MUST score, blood tests(Serum albumin (LFT’s), FBC, U&E’s) - Barriers to discharge identified - All oesophagectomy patients will have jejunostomy feeding, as well as selected gastrectomy patients. - Appropriate patients approached for entry into ethically approved studies |

**2.**

| **Pre-admission Counselling** |
| --- |
| - EROS pathway explained (patient and carers) - Patient information booklet provided - Anaesthetic assessment for all patients - Physiotherapy screening tool. - Day of surgery admission for all suitable patients who have been anaesthetically pre-assessed - Review of baseline nutritional assessment to define and counsel patients who require pre-operative feeding via TPN or jejunostomy - Counselling for patients who will have jejunostomy feeding, together with explanation that they will be discharged home with feeding tube in situ and that they may be discharged home with arrangements for home jejunostomy feeding. - Barriers to discharge identified - Prescribe 2 x 200ml nutricia ‘pre-op’ cartons to drink before 06:00 on the day of surgery - Check that patients have entered into ethically approved studies within the research portfolio – if not, why not? |
|
|
|
|
|
|
|
|

**3.**

| **Pre-operative Nutrition** |
| --- |
| - Normal diet 24 hrs before surgery. - No solid diet from 22.00 day before surgery. - Clear water up until 2 hrs before surgery. - 2 x Nutricia “PreOp” 200ml cartons to drink 06.00 day of surgery (unless dysphagia precludes this) |
|
|
|
|
|
|
|
|

**4.**

| **Pre-medication** |
| --- |
| - See “Drugs on Day of Surgery” guidelines. - Proton Pump Inhibitor (PPI) - No routine sedative pre-meds. |

**5.**

| **Anaesthesia** |
| --- |
| - Remit of individual upper GI anaesthetist - (But see points 9 and 11 below) |

**6.**

| **Analgesia** |
| --- |
| - Epidural/regional anaesthesia - Routine standard of care - Epidural - aim to remove on post operative day 3 - Regular paracetamol (initially IV, converted to oral as soon as possible) |

**7.**

| **Incision** |
| --- |
| - Laparoscopic procedures (transverse incisions if possible) - Open procedure - wound catheters and/or regional blocks |

**8.**

| **Nasogastric tubes** |
| --- |
| - Routine post-operative alimentary tract decompression for first 48 hours. - If BIRD considered as NIV respiratory support then nasogastric tube (NGT) needs to be in position on free drainage. If no NGT in place and NIV considered then contact oesophagogastric consultant prior to NIV |

**9.**

| **Fluid management** | |
| --- | --- |
| Pre-op | As above |
| Intra-op | Aim for goal directed fluid therapy, ideally with Doppler / LiDCO monitoring if available, to deliver a normovolaemic patient to recovery at the end of the procedure. |
| Post-op. | - 1^st^ 6 hrs of iv fluid prescribed by anaesthetist from recovery - Ongoing maintenance iv fluid –   0.18% / 4% dextrose-saline + 2g KCL at 1mg/kg/hr   - Stop ivi when tolerating 800ml oral fluid / day - Aim urine output 0.33 ml /kg/hr averaged over a 4 hr period. |

**10.**

| **Blood Tests** |
| --- |
| - Check FBC, U&E’s (inc Mg), LFT’s & CRP daily from POD1 to POD7 - Continue blood tests as clinically indicated. |

**11.**

| **Chest Drains** |
| --- |
| - Chest drain(s) will be placed at time of surgery to drain the pleural space. If more than one chest drain is placed the 2 drains will be clearly labelled where inserted, and this labelling will also be applied to the fluid balance chart - Drainage from chest drains needs to be documented on the fluids chart at 7am every day. The chart should record the total volume drained over the preceding 24 hour period. Documentation in this way means that informed decisions regarding drain removal can be made on daily 8am ward round - Drains will generally be removed on 3^rd^ or 4^th^ post operative day - For drain to be removed, drainage will need to be less than 150ml per 24 hours and drain fluid will need to be clear with no evidence of chyle - Chest X Ray should be performed within 1 hour of chest drain removal in order to confirm continued expansion of lung within chest. Confirmation that chest xray has been performed and reviewed by doctor to be documented in pathway |

**12.**

| **Nutrition** |
| --- |
| - All patients having resection will have been assessed by dietician at or before pre-assessment and a plan for their post operative nutritional pathway will have been made and documented prior to admission for surgery. - Nutritional pathways:   i) Oral enteral nutrition  All Oral nutrition post op will follow the feeding policy post oesophagectomy or major gastric resection (available on staffnet)  ii) Jejunostomy feeding  The purpose of jejunostomy feeding is to provide enteral nutrition when it is anticipated that patients may be unable to proved adequate enteral nutrition by mouth in the initial post-operative period to the extent that this may compromise their post operative recovery. There are two feeding regimens for jejunostomy feeding, and the regimen to be followed will generally be determined pre-admission.    a) Standard regime  b) Refeeding regime  iii)TPN  TPN regime will be as prescribed on individual basis  iv)Oral nutrition in patients with feeding jejunostomy  For post-operative days 1-4, patients will be progressed according to the hospital feeding policy post-major upper GI resection. Contrast swallow will be performed in order to confirm adequate emptying of the gastric tube.  If contrast study (and NUNscore) is satisfactory patients will be progressed to step 3 and jejunostomy reduced over following 3 days prior to discharge.  If contrast study shows poor emptying/function of gastric tube patients will be built up to full rate jejunostomy feeding and remain on step 2 of feeding pathway. Arrangements will be made for discharge home on day 8 with home jejunostomy, with training of patient on ward, provision of feed and feeding pump. Patients will build up oral intake at home and reduce jejunostomy feed under supervision of dietician and surgical team. |

**13.**

| **Bladder Catheterisation** |
| --- |
| - Hourly urine measurements first 48 hours - Aim to remove on POD 3-5 |

**14.**

| **Enhanced mobilisation** |
| --- |
| - Early and sustained mobilisation is key to success. - Mobilisation immediately post-op as set out below will require significant support and encouragement with the aim for independent mobilisation at POD 5 - On day of surgery sit out of bed for 2 hrs - POD 1 – mobilise 25-50m x 2 - POD 2 – mobilise 25-50m x 3 - POD 3 – mobilise 25-50m x 4 - POD 4 – mobilise 50-100m x 4 - POD 5 – mobilise 50-100m x 5 - POD 6 – mobilise 50-100m x 6 - POD 7 – mobilise 50-100m x 6 - POD 8 – continued mobilisation at home |

**15.**

| **Discharge criteria** |
| --- |
| - Observations and routine bloods within normal ranges. - Tolerating pureed diet Step 4 as in feeding policy. - If on home jejunostomy feeding, equipment arranged and training completed - Independently mobile - EROS Hotline number given in case of emergency. - Post op instructions on care, feeding etc given to patient and relatives - Follow up outpatient appointment 2 weeks post discharge made and given to patient |

16.

| **Post Discharge Contact and Follow Up** | |
| --- | --- |
| Contact Mobile Held By: | - EROS co-ordinator (8am-4pm weekdays) - E8 Nurse in charge (4pm-9pm weekdays, 8am-9pm weekends) - Night sister (9pm-8am 7 days a week) |
| - Telephone follow-up by EROS co-ordinator at day 1 and 3 post discharge. | |
| - Patients may contact the EROS team for up to 2 weeks post surgery. - Phone call to patient at 1 and 2 weeks post discharge by EROS co-ordinator. If well discharged from EROS programme. - Follow up out patient appointment 2 weeks post discharge. | |
| - Out of hours service notified of all EROS discharges as vulnerable patients to ensure timely assessment out of hours, by nursing staff at time of discharge. | |

**17.**

| **Re-admission Criteria** |
| --- |
| Same day assessment: |
| - Symptoms of sepsis – unwell, fever, - Cardio-pulmonary symptoms – Chest pain, breathlessness, leg swelling - Abdominal symptoms – Abdominal pain >2hrs, bloating, vomiting |
| Next day assessment: |
| Wound pain, redness, discharge |
| - Patients are assessed on F6 acute surgical admissions unit. They do not need to be seen by a GP prior to readmission. - Struggling nutritionally. |

**18.**

| **Audit** |
| --- |
| **Integral to EROS programme:**   - Morbidity / mortality (body part severity and Clavian Dindo classification) - Length of hospital stay - Re-admission rate - Day 7 POMS |

**EROS DAILY GOALS**

| **Evening post-surgery** |  |
| --- | --- |
| **General management** | - MEWS observations, according to patient observations - Prescribed Thomboprophylaxis 2 hours   post surgery. (According to protocol)   - TEDS stockings - PPI |
| **Fluid balance** | - IV maintenance dextrose-saline + 2gKCL at 1ml/kg/hr - Hourly catheter measurements - Maintain 0.33 ml/kg/hr (ave. over 4 hrs) |
| **Nutrition** | - Step 1 - Pink sticks/mouth care only |
| **Analgesia** | - Epidural/PCA - Regular paracetamol 1g tds IV - Anti-emetic prescribed |
| **Chest** | - Deep breathing exercises - Encourage cough |
| **Mobility** | - Sit out of bed 2 hrs - Circulatory exercises - Flowtron boots |
| **Jejunostomy** | - Water at 10ml/hour |
| **Chest Drain** | - Check chest drain(s) swinging |

| **Day 1 post-surgery** |  |
| --- | --- |
| **General management** | - Check U&E’s and FBC - MEWS observations - Thromboprophylaxis as per Trust Guidelines - PPI - Inspect wound dressing. (Wound intact, no signs of infection) |
| **Fluid balance** | - Step 1 – mouth care/pink sticks only - iv maintenance dextrose-saline + 2gKCL at 1ml/kg/hr Stop ivi if oral intake >800mls. - Hourly catheter measurements - Urine output - maintain 0.33 ml/kg/hr (average over 4 hrs) - Chart chest drainage – completion of surgery to 7am and |
| **Nutrition** | - Step 1 - Pink sticks/mouth care only |
| **Analgesia** | - Epidural - Regular paracetamol 1g tds - Anti-emetic prescribed |
| **Chest** | - Routine chest physiotherapy - Deep breathing exercises - Encourage cough. |
| **Mobility** | - Sit out of bed 4 hrs - Circulatory exercises - 2 walks 25-50m |
| **Jejunostomy** | - Start feed as per regimen |
| **Chest Drain** | - Document drainage at 7am mark bottle with time/date |

| **Day 2 post-surgery** |  |
| --- | --- |
| **General management** | - MEWS observations - Thromboprophylaxis as per Trust Guidelines. - TEDS stockings - PPI - Inspect wound dressing. (Wound intact, no signs of infection) |
| **Fluid balance** | - Step 2 - Sips of water/black tea or coffee (max 50mls/Hr) - iv maintenance dextrose-saline + 2gKCL at 1ml/kg/hr Stop ivi if oral intake >800mls. |
| **Nutrition** | - Step 2 - up to 60 ml/hour clear fluids |
| **Analgesia** | - Epidural/PCA - Regular oral paracetamol 1g qds - Regular NSAID if no contraindication - Anti-emetic prescribed |
| **Chest** | - Routine chest physiotherapy - Deep breathing exercises - Encourage cough |
| **Mobility** | - Sit out of bed 6 hrs - Circulatory exercises - 3 walks, 25-50m. |
| **Jejunostomy** | - Increase feed to 30ml per hour |
| **Chest Drain** | - Document drainage at 7am |

| **Day 3 post-surgery** |  |
| --- | --- |
| **General management** | - MEWS observations - Check U&E’s FBC - Thromboprophylaxis as per Trust Guidelines - TED stockings - PPI - Inspect wound dressing. (Wound intact, no signs of infection) |
| **Fluid balance** | - Step 2 - Sips of water/black tea or coffee (max 50mls/Hr) - iv maintenance dextrose-saline + 2gKCL at 1ml/kg/hr Stop ivi if oral intake >800mls. - Remove urinary catheter |
| **Nutrition** | - Step 2 – up to 60ml/hour clear fluids |
| **Analgesia** | - Stop PCA - Regular oral paracetamol 1g qds - Regular NSAID if no contraindication |
| **Chest** | - Deep breathing exercises - Encourage cough. |
| **Mobility** | - Self caring - Sit out of bed 6 hrs - Circulatory exercises - 4 ward circuits. |
| **Discharge** | - Discharge criteria fulfilled - Fit for discharge |
| **Jejunostomy** | - Increase feed to 50ml per hour |
| **Chest Drain** | - Document drainage at 7am |

| **Day 4 post-surgery** |  |
| --- | --- |
| **General management** | - Oral contrast study - to confirm adequate function and emptying of gastric tube - MEWS observations - Thromboprophylaxis as per Trust Guidelines - TED stockings - PPI - Bloods taken for NUN score calculation: (FBC, albumin and CRP) - Nun score results:   <10 progress according to protocol  >10 do not progress to free fluids unless discussed with consultant surgeon in all cases.   - Inspect wound dressing. (Wound intact, no signs of infection) |
| **Fluid balance** | - Fluids as tolerated - Chart fluid balance |
| **Nutrition** | IF Contrast study satisfactory AND NUN score <10 – home oral feeding pathway   - Step 3 - 3 oral nutritional supplement drinks   (Fortisip / Fortijuice)  IF contrast study suggests poor gastric emptying – home jejunostomy pathway   - Build up jejunostomy - Remain on step 2 orally |
| **Analgesia** | - Regular oral paracetamol 1g qds - Regular NSAID if no contraindication |
| **Chest** | - Deep breathing exercises - Encourage cough. |
| **Mobility** | - Self caring - Sit out of bed 6 hrs - Circulatory exercises - 5 ward circuits. |
| **Jejunostomy** | - Increase feed at 70ml per hour if still on step 2 |
| **Chest Drain** | - Document drainage at 7am - Consider removal if <150ml |

| **Day 5 post-surgery** |  |
| --- | --- |
| **General management** | - MEWS observations - Thromboprophylaxis as per Trust Guidelines - TED stockings - PPI - Remove wound dressing. (Wound intact, no signs of infection) |
| **Fluid balance** | - Free fluids as tolerated - Chart fluid balance |
| **Nutrition** | Home oral feeding pathway   - 3 oral nutritional supplement drinks   (Fortisip / Fortijuice)   - Step 4 - Progress to Puree C diet   Home Jejunostomy feeding pathway   - Step 2 – 60ml per hour clear fluids - Jejunostomy feed – see below |
| **Analgesia** | - Regular oral paracetamol 1g qds - Regular NSAID if no contraindication |
| **Chest** | - Deep breathing exercises - Encourage cough. |
| **Mobility** | - Self caring - Sit out of bed 6 hrs - Circulatory exercises - 6 ward circuits. |
| **Jejunostomy** | Run feed at full rate  Commence training for home jejunostomy feed if planned  Liaise with dietician to confirm arrangements in place for home pump/supervision |
| **Chest Drain** | - Document drainage at 7am - Consider removal if <150ml |
| **Discharge** | - Confirm discharge plan in place: - Confirm likely to be fit for discharge. - Identify barriers to discharge |

| **Day 6 post-surgery** |  |
| --- | --- |
| **General management** | - QDS MEWS observations - Thromboprophylaxis as per Trust Guidelines - TED stockings - PPI - Check wounds |
| **Fluid balance** | - Chart fluid balance |
| **Nutrition** | Home oral feeding pathway   - 3 oral nutritional supplement drinks   (Fortisip / Fortijuice)   - Step 4 - Progress to Puree C diet   Home Jejunostomy feeding pathway   - Step 2 – 60ml per hour clear fluids - Jejunostomy feed – see below |
| **Analgesia** | - Regular oral paracetamol 1g qds - Regular NSAID if no contraindication |
| **Chest** | - Deep breathing exercises - Encourage cough. |
| **Mobility** | - Self caring - Sit out of bed 6 hrs - Circulatory exercises - 6 ward circuits. |
| **Jejunostomy** | - Run feed at full rate - Continue training for home jejunostomy feed |
| **Chest Drain** | - Document drainage at 7am - Consider removal if <150ml - If chest drain not removed, at this stage discuss with consultant |
| **Discharge** | - Discharge plan in place - - confirm patient and carers understand - verbal and written information - Edocs discharge summary written in draft - Barriers to discharge identified, action plan to manage |

| **Day 7 post-surgery** |  |
| --- | --- |
| **General management** | - QDS MEWS observations - Thromboprophylaxis as per Trust Guidelines - TED stockings - PPI |
| **Fluid balance** | - Free Fluids |
| **Nutrition** | Home oral feeding pathway   - 3 oral nutritional supplement drinks   (Fortisip / Fortijuice)   - Step 4 - Progress to Puree C diet   Home Jejunostomy feeding pathway   - Step 2 – 60ml per hour clear fluids - Jejunostomy feed – see below |
| **Analgesia** | - Regular oral paracetamol 1g qds - Regular NSAID if no contraindication |
| **Chest** | - Deep breathing exercises - Encourage cough. |
| **Mobility** | - Self caring - Sit out of bed 6 hrs - Circulatory exercises - 6 ward circuits. |
| **Discharge** | - Confirm discharge plan - Ensure POMS proforma completed - Diet to be followed at home – confirm information has been given and that this is clear to patient and carers - E-docs discharge summary written - TTOs prescribed and ordered from pharmacy |
| **Day 8 post-surgery** |  |
| **General management** | - No MEWS observations - Thromboprophylaxis as per Trust Guidelines - TED stockings - PPI - Final wound check |
| **Fluid balance** | - Free fluids |
| **Nutrition** | Home oral feeding pathway   - 3 oral nutritional supplement drinks   (Fortisip / Fortijuice)   - Step 4 - Progress to Puree C diet   Home Jejunostomy feeding pathway   - Step 2 – 60ml per hour clear fluids - Jejunostomy feed – see below |
| **Analgesia** | - Regular oral paracetamol 1g qds - Regular NSAID if no contraindication |
| **Chest** | - Deep breathing exercises - Encourage cough. |
| **Mobility** | - Self caring - Sit out of bed 6 hrs - Circulatory exercises - 6 ward circuits. |
| **Jejunostomy** | - Patient or carers to be signed off as competent with pump/feed etc. - Ward to organise 7 day supply of feed/sterile water/giving sets and syringes |
| **Discharge** | - Home |

# Southampton Dieticians’ Enteral Feeding Nutrition Chart

EROS STANDARD STARTER REGIMEN

| Patient name: ___________________________ D.o.b. ___________________ Hospital No: ­­­­­­­­­­­­­­________________ **Feeding route: __________________________ Weight: ____________ kg**  **Estimated/weighed** (circle as appropriate) |
| --- |

**☑ Use giving set & reservoir as per manufacturers instruction**

**☑ Avoid decanted feeds, but if used do not hang for more than 4 hours**

**☑ Flush the feeding tube with 30ml of sterile water before and after feed or medication is given using a 50ml bladder syringe**

**☑ Additional fluid may be required. Please discuss with Medical team**

**☑ Keep head elevated to 30-45º during and shortly after feeding**

**☑ Monitor bowels**

**☑ Keep head elevated to 30-45º during and shortly after feeding**

**☑ If Potassium (K), Magnesium (Mg), Phosphate (PO_4_) are low do not increase feed rate – do inform Medical team.**

| **Date** | **Feed/Water** | **Volume per 24 hrs** | **Rate ml/hr** | **Hours of**  **Feeding** | **Rest**  **Hours** | **Total energy, protein & fluid** |
| --- | --- | --- | --- | --- | --- | --- |
| **DAY (1)** | **NUTRISON 1.0**  **(NUTRISON STANDARD)** | **600ml** | **25ml/hr** | **24 hours** | **0 hours** | **600kcal, 24g protein, 600ml** |
| **DAY (2)** | **NUTRISON 1.0**  **(NUTRISON STANDARD)** | **1200ml** | **50ml/hr** | **24 hours** | **0 hours** | **1200kcal, 48g protein, 1200ml** |

**PLEASE REFER PATIENT TO DIETICIAN**

# Southampton Dieticians’ Enteral Feeding Nutrition Chart

**FOR PATIENTS AT RISK OF RE-FEEDING SYNDROME**

EROS STARTER REGIMEN FOR PATIENTS AT RISK OF RE-FEEDING SYNDROME

| Patient name: ___________________________ D.o.b. ___________________ Hospital No: ­­­­­­­­­­­­­­________________ **Feeding route: __________________________ Weight: ____________ kg**  **Estimated/weighed** (circle as appropriate) |
| --- |

**☑ Use giving set & reservoir as per manufacturers instruction**

**☑ Avoid decanted feeds, but if used do not hang for more than 4 hours**

**☑ Flush the feeding tube with 30ml of sterile water before and after feed or medication is given using a 50ml bladder syringe**

**☑ Additional fluid may be required. Please discuss with Medical team**

**☑ Keep head elevated to 30-45º during and shortly after feeding**

**☑ Monitor bowels**

**☑ Ensure re-feeding vitamins are prescribed – refer to re-feeding guidelines on Staffnet**

**☑ Ensure Potassium (K), Magnesium (Mg) and Phosphate (PO_4_) are checked daily until stable**

**☑ If K, Mg, PO_4_ are low do not increase feed rate – do inform Medical team**

| **Date** | **Feed/Water** | **Volume per 24 hrs** | **Rate ml/hr** | **Hours of**  **Feeding** | **Rest**  **Hours** | **Total energy, protein & fluid** |
| --- | --- | --- | --- | --- | --- | --- |
| **DAY (1)** | **NUTRISON 1.0**  **(NUTRISON STANDARD)** | **240ml** | **10ml/hr** | **24 hours** | **0 hours** | **240kcal, 9.6g protein, 240ml** |
|  | **If K, Mg, PO4 checked and stable/supplemented, progress to Day 2** |  |  |  |  |  |
| **DAY (2)** | **NUTRISON 1.0**  **(NUTRISON STANDARD)** | **480ml** | **20ml/hr** | **24 hours** | **0 hours** | **480kcal, 19.2g protein, 480ml** |

**PLEASE REFER PATIENT TO THE DIETICIAN**
